# STING orchestrates microglia polarization via interaction with LC3 in autophagy after ischemia

**DOI:** 10.1038/s41419-024-07208-1

**Published:** 2024-11-13

**Authors:** Lingqi Kong, Pengfei Xu, Nan Shen, Wenyu Li, Rui Li, Chunrong Tao, Guoping Wang, Yan Zhang, Wen Sun, Wei Hu, Xinfeng Liu

**Affiliations:** 1 Department of Neurology, The First Affiliated Hospital of USTC, Division of Life Sciences and Medicine, University of Science and Technology of China, Hefei, Anhui 230001 China; 2 School of Life Sciences, Division of Life Sciences and Medicine, University of Science and Technology of China, Hefei, Anhui 230026 China; 3 Department of Neurology, Centre for Leading Medicine and Advanced Technologies of IHM, The First Affiliated Hospital of USTC, Division of Life Sciences and Medicine, University of Science and Technology of China, Hefei, Anhui 230001 China

**Keywords:** Microglia, Stroke

## Abstract

Autophagy has both protective and pathogenetic effects on injury caused by cerebral ischemia/reperfusion (I/R). Our previous research has indicated that stimulator of interferon genes (STING) could orchestrate microglia polarization following middle cerebral artery occlusion. However, it remains largely unexplored whether STING balances microglial polarization by regulating autophagy in brain I/R injury. Here, STING was observed to show an up-regulation in the microglia from mice subjected to experimental ischemic stroke. Strikingly, the deletion of STING led to the significant skewness of microglia activated by ischemia from a pro- to anti-inflammatory state and substantially alleviated ischemia-induced infarction and neuronal injury. In addition, STING-null mice can restore long-term neurobehavioral function. Then, the crosstalk between neuroinflammation and microglia autophagy was analyzed. The differential activity of autophagy in wild-type and STING-knockout (KO) mice or primary microglia was largely reversed when STING was restored in microglia. Irritating autophagy by rapamycin skewed the anti‑inflammatory state induced by STING-KO to a pro‑inflammatory state in microglia. Furthermore, microtubule-associated protein light-chain-3 (LC3) was identified as the key factor in the STING regulation of autophagy by glutathione-S-transferase (GST) pull-down analysis. Mechanically, STING can directly interact with LC3 through the STING transmembrane domain (1-139aa). Herein, current data determine the pivotal role of autophagy, specifically via LC3 protein, in the regulation of microglial phenotypic transformation by STING. These findings may provide a possible treatment target for delaying the progression of ischemic stroke.

## Introduction

As a most common neurological disease, stroke affects more than 13.7 million people each year [1]. Ischemic stroke accounts for approximately 70% of stroke cases because the rupture or blockage of blood vessels leads to insufficient blood supply [2]. Nevertheless, treatment options for stroke are quite limited. Although the acute care phase can rely on thrombolytic treatment, a number of ischemic patients miss the opportunity for thrombolytic therapy due to the therapeutic time window of less than 6 h [3, 4]. Moreover, the restoration of blood supply will further exacerbate the progression of brain injury, a process known as ischemia/reperfusion (I/R) injury [5]. This adds to the complexity of the pathophysiology of ischemic stroke. During the whole process, a crucial element is the immune system, especially macrophages in brain parenchyma, namely microglia [6].

Microglia, the main resident immune cells of the central nervous system (CNS), rapidly respond to cerebral ischemic injury [7, 8]. It is universally accepted that microglia serve as a double-edged sword of brain immune response [6, 9]. Activated microglia include classical (aggravate inflammation and neuronal dysfunction) and alternative (favor tissue repair and increase neuronal survival) phenotypes [6, 9, 10]. Microglia experience unique spatial-temporal changes under pathophysiological conditions, which suggests the indispensability of each microglial phenotype in ischemic stroke [8, 11]. Therefore, cerebral I/R injury is likely to be prevented or reduced by exploring novel immunomodulatory strategies that balance microglial phenotypes in the acute ischemic penumbra.

Autophagy is a highly conservative process of intracellular degradation that removes dysfunctional or unwanted proteins and organelles, as well as invading pathogens, through autophagy-lysosome machinery [12–14]. In fact, autophagy balances beneficial and detrimental effects of immunity and inflammation [15–17]. Meanwhile, autophagy is linked to the inflammatory response induced by microglia after ischemia stroke and is crucial for microglia phenotype switching [18, 19]. For example, activating autophagy has been shown to increase the inflammatory responses of microglia under lipopolysaccharide (LPS) stress [20]. As noted by Yang et al., inhibiting autophagy resulted in a decrease in inflammatory injury and microglial activation in intracerebral hemorrhage mice [21]. Nonetheless, the activation of microglial autophagy was also observed to facilitate the M2-phenotype polarization [22, 23]. Overall, autophagy may be characterized by a balance between pro- and anti-inflammatory microglia, but the specific mechanism remains elusive.

Interestingly, the stimulator of interferon genes (STING) is mainly expressed in microglia and acts with cyclic GMP-AMP synthase (cGAS) to recognize damage-associated molecular patterns (DAMPs) [24, 25]. Autophagy induction is a basic feature of the cGAS-STING pathway [26]. A previous study reported that STING activation interferes with autophagy in an interferon (IFN)-dependent manner to aggravate lung injury [27]. Moreover, the cGAS-STING pathway has drawn special attention in stroke, subarachnoid hemorrhage (SAH) and other inflammatory CNS diseases [28–30]. Our previous study showed that STING aggravated ischemic brain injury by facilitating microglia towards M1 modality [31]. However, the involvement of autophagy and the underlying mechanisms remain unclear. This study was aimed at investigating the underlying role of STING in autophagy and the pathological process of cerebral I/R injury. According to the present data, STING facilitated autophagy in mouse brains and primary microglia, and pro-inflammatory microglia phenotype was significantly reversed to anti-inflammatory microglia phenotype through ablation of STING. This reveals the activation of autophagy in the phenotype transformation of microglia induced by STING.

## Materials and methods

### Animal

Offered by GemPharmatech LLC (Jiangsu, China), male *Sting1*-knockout (KO) (B6/JGpt-*Sting1*^*em10Cd12452*^/Gpt, Strain NO. T012747) and C57BL/6J wild-type (WT) mice were used at 6–8 weeks of age and 20–25 g of weight. They were kept in specific pathogen-free equipment with a 12 h light/dark cycle, controlled humidity and temperature, and free access to water and food in plastic cages. Every procedure gained the approval of the Animal Ethics Review Committee of The First Affiliated Hospital of the University of Science and Technology of China. The implementation of experimental protocols followed the National Institute of Health Guide for the Care and Use of Laboratory Animals (NIH Publications No. 80-23, revised 1996). In each experiment, mice adapted themselves to the environment for 1 week before being randomly assigned to individual groups. The experimental design is shown in Fig. S1. Utmost efforts were made to reduce the number and pain of animals.

### Mouse middle cerebral artery occlusion model

The procedures of establishing a middle cerebral artery occlusion (MCAO) model were described before [32]. Isoflurane was used to anesthetize mice whose common carotid, external carotid and internal carotid arteries (CCA, ECA and ICA, respectively) were surgically exposed through a midline neck incision. A silicon-coated nylon suture (Beijing, Cinontech Co., Ltd., China) was inserted into the ICA through the ECA stump to occlude the MCA. Then, 90 min of occlusion was followed by the withdrawal of the suture to allow reperfusion. Laser Speckle Doppler Flowmetry (PeriCam PSI Z; Perimed, Sweden) was utilized to monitor cerebral blood flow (CBF) during surgery. This study included mice that had a reduction >75% of baseline in the ischemic core (Fig. S2) and excluded those that died during surgery. The same procedure was performed on Sham-operated mice, except that the nylon monofilament was inserted. An electric heating blanket was used to maintain the body temperature of mice until their complete recovery.

### Cell culture and hypoxic treatment

The culture of primary microglia cells was previously described [33, 34]. Mixed glial cells from one- to three-day-old WT or STING^−/−^ pups were built and grown in Dulbecco’s Modified Eagle’s Medium (DMEM, Gibco) with 1% penicillin-streptomycin (Gibco, USA) and 10% fetal bovine serum (Gibco, USA) for 14 days. A shaking method was employed to obtain high-purity microglia that were then seeded onto plates after the 14-day maintenance of the mixed glial culture. In vitro, a glucose-free DMEM (Gibco, USA) was utilized to replace the cell culture medium to induce hypoxia according to previous methods [32, 33]. Afterwards, the culture was incubated in a sealed chamber armed with AnaeroPack-Anaero (Mitsubishi Gas Chemical Co., Inc., Japan) at the temperature of 37 °C. Primary microglia cells received oxygen-glucose deprivation (OGD) for 8 h and were then put back into the normal incubator with their maintenance medium for reoxygenation. A normoxic incubator was adopted to culture control cells for the same period.

### Drugs and treatment

Mice were intraperitoneally injected with rapamycin (10 mg/kg body weight) (MedChem Express, MCE) or equivalent volumes of vehicle for three consecutive days after MCAO surgery [35]. In vitro, 100 nM rapamycin or vehicle was applied to treat primary microglia cells for 24 h [20, 35]. Primary microglia were stimulated with 100 μg/ml 5,6-dimethylxantheonone-4-acetic acid (DMXAA, MedChem Express, MCE) for 24 h [36]. The determination of dose and duration was based on previous studies and the manufacturer’s established protocols. Control cells were maintained under normal conditions.

### Stereotaxic injection and viral infection

Adeno-associated virus (AAV) vectors that encode STING and scramble (negative control, NC) were established by GeneChem (Shanghai, China). STING was specifically expressed in microglia by producing a sequence encoding STING and the enhanced green fluorescent protein (EGFP) reporter gene (AAV-F4/80-MCS-EGFP-3Flag-SV40 PolyA) and inserting it into serotype 9 AAV packaging vectors. The right hemisphere was injected with packaged AAV using stereotaxic injection (dosage of 3 μl; coordinates: −0.23 mm anterior-posterior, −1.0 mm medial-lateral, −2.6 mm dorsal ventral relative to the bregma). The injection was conducted 2 weeks before the surgery. Transfection efficiency was measured by immunostaining.

As per the protocol of the manufacturer, AAV-mCherry-GFP-LC3B was purchased by Beyotime (C3013, Beyotime, China) and used for autophagy detection after stereotaxic injection into mouse brains 2 days before MCAO. The injection coordinates were the same as those mentioned above. In vitro, primary microglia cells were injected with Lenti-mCherry-EGFP-LC3B (C3002, Beyotime, China) according to the instructions of the manufacturer.

Primary microglia were grown in plates with 96 wells at a density of 5 × 10^4^ cells and then transfected recombinant adenovirus (Adv) expressing EGFP Adv-LC3B-319, Adv-LC3B-320, Adv-LC3B-321 or Adv-NC (GeneChem, Shanghai, China) at various multiplicities of infection (MOIs). Transduction efficiency was evaluated by fluorescence microscopy at 6, 12 and 24 h after transfection. The optimal MOI was determined by western blot for LC3 gene expression.

### Behavior tests

#### Modified neurological severity score

Motor coordination and dysfunction were tested by selecting a modified neurological severity score (mNSS), as described before [11, 31, 32].

#### Accelerating rotarod test

For the rotarod test, mice were placed on a rotating rod, whose speed increased from 4 to 40 rpm/min within 2 min. Mice were trained for three days before surgery, and the speed data on the final day of training was recorded as the baseline value. [37]. Then, mice were tested 3, 7, 14 and 21 days after injury, and the latency to fall was analyzed.

#### Novel object recognition test

A novel object recognition (NOR) test was carried out to evaluate hippocampus-dependent memory deficits as previously described [38, 39], followed by the habituation of mice in an empty open field box (40 cm × 40 cm). Mice were permitted to freely identify two identical objects placed in the corner of the chamber for 5 min before being returned to plastic cages. After each trial, 75% ethanol was used to clean the box and objects. On the test day, one object was replaced with a novel object, and the mice explored again. The percentage of exploratory preference was calculated by the software Smart Video (Panlab Harvard Apparatus, Spain).

#### Y-maze

Spontaneous alternation in a Y-maze was conducted to assess spatial working memory as previously described [39, 40]. The three arms of the Y-maze were marked as distinguishable identifiers. Mice were removed and gently placed into an arm of the Y-maze to face the center. The spontaneous behavior of each mouse for 5 min was recorded, and spontaneous alternation (%) was then calculated as: number of arm alternations / (total number of arm entries − 2) × 100%.

#### Morris water maze test

The Morris water maze (MWM) test, which began on the 23rd day after surgery, was used to measure long-term cognitive changes [31, 38]. Briefly, on the first five days, the mice were released from four different quadrants and trained to look for the platform for 60 sec per day. During the probe phase, all mice were placed into the water maze for the 60 sec exploration of the platform after the removal of the platform. Latency time, swim path length, the time in the platform zone and the number of platform crossovers were analyzed using Smart-tracking software (Panlab Harvard Apparatus, Spain). The procedure of evaluation was performed by a researcher blind to the group identity of mice.

### Infarct and edema volume measurements

The 2,3,5-triphenyltetrazolium chloride (TTC; Sigma Aldrich Inc., USA) assay was performed to determine infarction volume after MCAO [41]. After being removed, mouse brains were cut into coronal sections in a brain mold. These coronal sections underwent 15 min incubation with TTC staining solution at 37 °C before being saved in 4% formalin solution. A researcher blinded to group conditions utilized a scanner (Epson Perfection V19 Scanjet; Seiko Epson, Japan) to obtain images and calculated these images using Image J software (NIH, Bethesda, MD, USA) for the measurement of infarct and edema volumes. The formulas for calculation are as follows: infarct volume ratio = [(contralateral brain volume – ipsilateral non-infarcted brain volume) / contralateral brain volume × 2] × 100%, edema ratio = (ipsilateral brain volume − contralateral brain volume) / contralateral brain volume × 100%.

### Immunofluorescence and staining

First, 0.9% saline was used to perfuse mouse brains, and then 4% paraformaldehyde was used to fix them. Brain tissues were completely dehydrated in 30% sucrose and then refrigerated in the Tissue-Tek O.C.T. compound (Sakura Finetek, USA). Sequential coronal slices were obtained with the microtome (CM1950; Leica Microsystems, Wetzlar, Germany). A solution with 0.1% Triton X-100, 10% donkey serum and 1% bovine serum albumin (BSA) was used to block cells or brain sections. Subsequently, cells or brain sections were incubated at 4 °C overnight with antibodies directed against STING (1:4000, 19851-1-AP, Proteintech), p-STING (1:2000, AF7416, Affinity Bioscience), ionized calcium-binding adapter molecule 1 (Iba1, 1:1000, 011-27991, Wako), cluster of differentiation 16/32 (CD16/32, 1:500, 553142, BD Biosciences), CD206 (1:200, AF2535, R&D Systems) and LC3B (1:1000, ab192890, CST). Next, the sections were cleaned in phosphate buffer solution (PBS) and incubated for 2 h using appropriate secondary antibodies at room temperature. Such secondary antibodies included donkey anti-mouse (H&L, Alexa Fluor® 488, ab150105, Abcam), -rabbit (H&L, Alexa Fluor® 488, ab150073, Abcam), -goat (H&L, Alexa Fluor® 594, ab150132, Abcam), -rat (H&L, Alexa Fluor® 647, ab150167, Abcam) and -rat immunoglobulin G (IgG) (H&L, Alexa Fluor® 594, ab150156, Abcam). After that, the sections were cleaned with PBS, followed by the staining of the nuclei with 40,6-diamidino-2-phenylindole (DAPI).

For the co-staining of NeuN and TUNEL, the sections were first stained with NeuN antibody (1:1000, ab104224, Abcam), and One-Step TUNEL Assay Kit (Beyotime, China) was used for TUNEL staining as per the protocol of the manufacturer.

Neuronal degeneration was assessed by Fluoro-Jade C (FJC, Millipore, USA) staining [31, 42]. The frozen sections of mouse brains were successively soaked in 1% sodium hydroxide / 80% ethanol solution, 70% ethanol as well as 0.06% potassium permanganate solution. Then, 0.0001% FJC solution was used for the incubation of these sections.

Monodansylcadaverine (MDC) (C3018S, Beyotime, China) was a fast and convenient fluorescent probe used for analyzing the autophagy process [43]. MDC was utilized for the 30-min staining of cell plates in a cell incubator at 37 °C, and then cell plates were cleaned with PBS three times and observed under a confocal fluorescence microscope.

Three brain slices of each mouse were examined, and two regions within the ischemic boundary cortex and striatum zone of each section were randomly selected and imaged using consistent imaging parameters. Images were obtained using a confocal laser scanning microscope (Olympus FV3000, Japan) and quantitatively analyzed using Image J software by an investigator blinded to the experimental design.

### Flow cytometric analysis of microglia

For brain tissues, each mouse was anesthetized and intracardially perfused with 0.9% cold saline. Peri-infarct tissues were collected, minced and suspended in PBS. The apoptotic fraction was observed by staining with Annexin-V conjugated with fluorescein isothiocyanate and propidium iodide (PI) using the Annexin-V/PI Apoptosis Detection kit (BD Biosciences, Franklin Lakes, New Jersey, USA). Apoptotic cells are referred to as Annexin-V^+^ cells, including early (Annexin V^+^ / PI^−^) and late (Annexin V^+^ / PI^+^) stages. Appropriate isotype controls were used, and single staining was used for fluorochrome compensation. The cells were incubated with Annexin-V/PI, washed with PBS twice, re-suspended in 200 μl PBS solution, assessed on the BD Fortessa flow cytometer (Franklin Lakes, USA) and analyzed by use of FlowJo software (FlowJo, version 10.8, Ashland, Oregon, USA).

### Electron microscopy

Mice were anesthetized and intracardially perfused with 4% paraformaldehyde and 2.5% glutaraldehyde. Peri-infarct tissues were collected, cut into coronal slices with a thickness of 1 mm^3^ and then fixed in 1% osmium tetroxide. The samples were cleaned with PBS and subjected to 24-h flooding with 2% uranyl acetate at 4 °C. Then, they were osmicated, dehydrated with ethanol, embedded in epoxy resin and sectioned into slices (UC-7, Leica, Germany). Uranyl acetate and lead citrate were used to stain the thin sections, which were then observed with an electron microscope (JEM-1400, JEOL, Japan).

### GST pull-down assay

For glutathione-S-transferase (GST) pull-down assay, pGEX-4T-1 GST vectors were used to construct GST-STING such as GST-STING (1-379aa), GST-STING-1 (1-139aa) and GST-STING-2 (140-379aa) expressed in E.coli BL21 (DE3) cells. The culture was expanded after the further purification of the obtained GST fusion proteins. The mixture of GST fusion proteins and beads was added with the lysates derived from cerebral ischemic penumbra, and then these lysates underwent 4-h incubation at 4 °C with shaking. The proteins that were eluted were detected by Coomassie brilliant blue staining and western blot.

### Quantitative real-time polymerase chain reaction

Total ribonucleic acid (RNA) was extracted from cells or ischemic boundary tissues by Trizol (Invitrogen, USA) and reverse-transcribed to complementary deoxyribonucleic acid (cDNA) with HiScript III RT SuperMix for qPCR kit (Vazyme, China). The AceQ qPCR SYBR Green Master Mix kit (Vazyme, China) were used for quantitative reverse transcription polymerase chain reaction (qRT-PCR) on Light Cycler 96 System (Roche, Switzerland). Supplementary Table 1 shows the sequences of the primers used. qRT-PCR was performed in triplicate, and three repeat values were averaged for each sample. The levels of messenger RNA (mRNA) relative to glyceraldehyde 3-phosphate dehydrogenase (GAPDH) were normalized.

### Western blot analysis

The deep anesthesia (5% isoflurane) and decapitation of mice were performed. Ipsilateral (ischemic) hemisphere tissues or the cultured cells were homogenized in RIPA lysis buffer (Beyotime, China) with protease inhibitor (Beyotime, China) for 60 sec using a freezing grinder (JXFSTPRP-CLN, Jing Xin, China) and then centrifuged at 14,000 g (4 °C, 10 min). NanoPhotometer-N50 (IMPLEN GMBH, Germany) was applied to quantify protein concentration. Then, equivalent amounts of protein were isolated by 8–12% sodium dodecyl sulfate-polyacrylamide gel electrophoresis (SDS-PAGE) and transferred onto polyvinylidene difluoride (PVDF) membranes. The same samples were run on separate gels (sister gels) to test different proteins with different molecular weights. Next, these PVDF membranes were blocked with 5% BSA and received overnight incubation at 4 °C with primary antibodies. Such primary antibodies included p-STING (1:2000, AF7416, Affinity Biosciences), STING (1:4000, 19851-1-AP, Proteintech), inducible nitric oxide synthase (iNOS, 1:1000, 18985-1-AP, Proteintech), Arginase-1 (Arg-1, 1:1000, #93668, CST), CD16/32 (1:500, 553142, BD Biosciences), CD206 (1:200, AF2535, R&D Systems), LC3B (1:1000, ab192890, CST), sequestosome 1 (SQSTM1/P62, 1:1000, P0067, Sigma), autophagy-related gene 7 (ATG7, 1:1000, 10088-2-AP, Proteintech), ATG5 (1:1000, ab108327, Abcam), Beclin-1 (1:1000, ab62557, Abcam) and β-Tubulin (1:2000, A12289, ABclonal). The membranes underwent 1-h incubation with proper horseradish peroxidase (HRP)-conjugated secondary antibodies (1:10000, Willget biotech, China) at room temperature. Bands were visualized by the Azure 500 system (Azure Biosystems, USA) with enhanced chemiluminescence (ECL) reagents (Willget Biotech, China) and quantified with the Image J software. Values obtained from densitometry of the target proteins were normalized to β-tubulin for the same samples.

### Protein-protein docking

Protein Data Bank (PDB): STING1 and MAP1LC3b were selected as the starting models for STING and LC3. Two models were fine-tuned to achieve feasible conformations after the addition of missing residues, guided by AlphaFold2 predictions. The segment spanning residues 1 to 139 in STING1 was pinpointed for engagement, whereas MAP1LC3b had no constraints. A preferred binding orientation was determined based on biological evidence and computational rankings. Following molecular dynamics simulations, the structure was analyzed using the PISA interface server. Molecular illustrations were produced with the aid of PyMOL software.

### Statistical analysis

Each experiment was randomized, and statistical analysis was conducted with Statistical Package for the Social Sciences (SPSS) 22.0 software (International Business Machines Corporation, Armonk, New York, USA). One-way analysis of variance (ANOVA) followed by Tukey’s post hoc test was utilized for comparing multiple groups. Two-way ANOVA followed by Tukey’s post hoc test was used for analyzing the escape latency and swimming path length in MWM tests. All data were expressed as mean ± standard deviation (SD). All tests were considered to show statistical significance at *P* < 0.05.

## Results

### Expression levels and cellular locations of STING under ischemic-hypoxic conditions

**Fig. 1:**
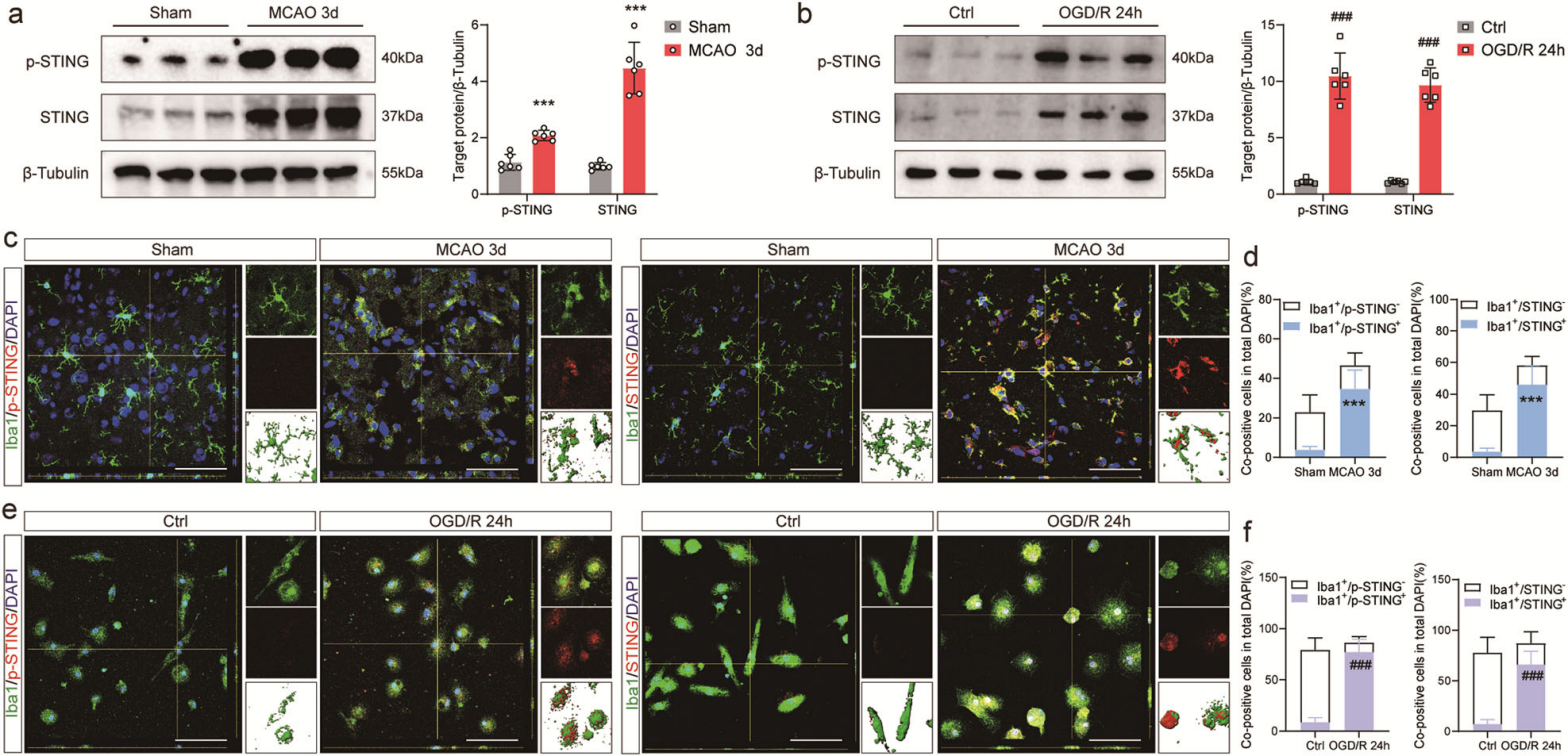
STING is activated after I/R and OGD/R. **a**, **b** Representative immunoblots and quantification of p-STING and STING in mouse ischemic penumbra and primary microglia cells at the indicated time points after I/R and OGD/R. STING, p-STING and Iba1 co-localization and quantification in MCAO mice 3 days after reperfusion (**c**, **d**) and in primary microglial cells 24 h after reoxygenation (**e**, **f**). Data are expressed as mean ± SD. *n* = 6. Scale bar = 50 μm. ^***^P < 0.001 vs Sham mice. ^###^*P* < 0.001 vs Ctrl microglia.

Considering the findings from our prior work [31], the 3rd day-time point was chosen for analyzing the expression levels and cellular locations of STING. The western blot results demonstrated that MCAO mice exhibited an obvious increase in p-STING and STING at 3 days after reperfusion compared with Sham-operated ones (Fig. 1a). Additionally, abundant p-STING and STING positive cells co-stained with Iba1 in the penumbral area 3 days after MCAO (Fig. 1c, d). Consistently, a significant increase in the expression levels of p-STING and STING was observed in primary microglia 24 h after reoxygenation, as evidenced by both western blot and immunofluorescence staining (Fig. 1b, e, f). The finding is consistent with previous studies where STING was mainly expressed in microglia 3 days post MCAO [44].

### Attenuation of infarct progression and neuronal injury by KO of STING

**Fig. 2:**
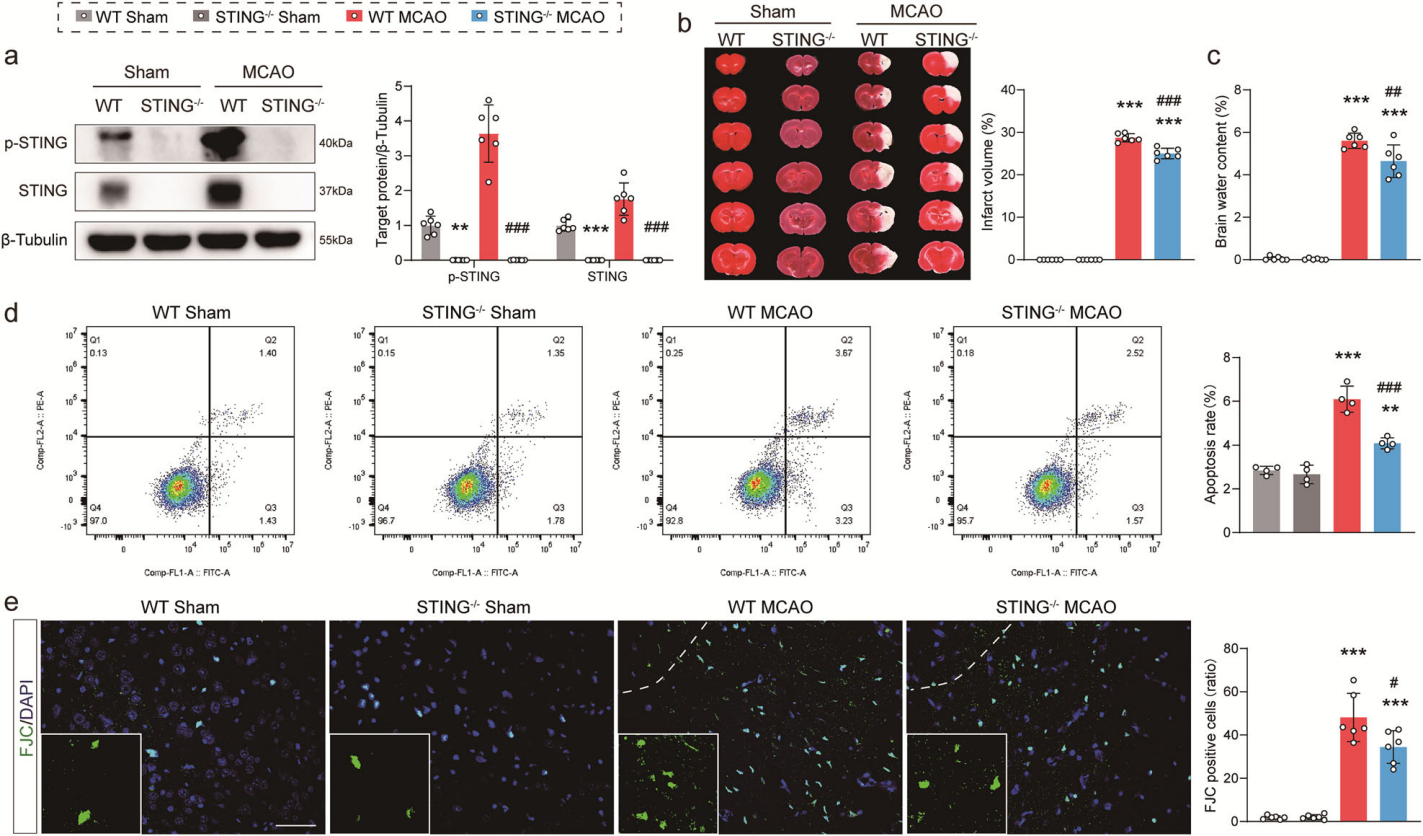
STING-null mice ameliorated brain infarction and neuronal damage following MCAO. **a** Protein levels of p-STING and STING in both WT and STING^−/−^ mice 3 days after MCAO and Sham-operated. **b** Infarct volume was assessed by TTC (2,3,5-triphenyltetrazoliumchloride) staining 3 days after the MCAO surgery. **c** Quantitative assessment of edema volume. **d** Flow cytometry analysis showing that compared with WT MCAO group, the reduction of apoptosis percentage following STING knockout conditions. **e** Representative images from peri-infart region stained with Fluoro-Jade C (FJC, green) and DAPI (blue), and insets show a higher magnification view. Analysis of the number of FJC-positive cells as a ratio of total cells. Data are expressed as mean ± SD. *n* = 6. Scale bar = 50 μm. ^**^P < 0.01, ^***^P < 0.001 vs Sham mice. ^#^*P* < 0.05, ^##^*P* < 0.01, ^###^*P* < 0.001 vs WT MCAO mice.

A mouse strain with the deletion of the STING gene was established. As shown in Fig. 2a, STING protein was not detected in the brain of STING-KO mice. In addition, whether the deletion of STING impaired development and movement in mice was checked. Body weight and reproductive state were recorded, and STING-KO mice were observed to be fertile and develop normally (Fig. S3a). The rotarod test (Fig. S3b) showed no significant differences in motor coordination and locomotor activity between WT and STING-KO mice.

Next, the biological function of STING in the MCAO model was explored. Compared with animals of the WT group, those of the STING^−/−^ group exhibited a significant reduction in brain infarct volume and edema at 3 days after MCAO surgery (Fig. 2b, c). The results of flow cytometry revealed that the WT MCAO group exhibited a marked increase in cellular apoptosis in comparison with the WT Sham group, while the ablation of STING can significantly reduce this trend (Fig. 2d). Moreover, the WT MCAO group showed a remarkable increase in FJC-positive stained cells in the ischemic penumbra compared with the WT Sham group. However, the STING^−/−^ MCAO group greatly decreased FJC-positive degenerated neurons in ipsilateral lesions compared to the WT MCAO group (Fig. 2e). These data indicated that STING is probably a key factor in poor neurological outcomes induced by ischemia.

### Amelioration of long-term neurobehavior function by the lack of STING

**Fig. 3:**
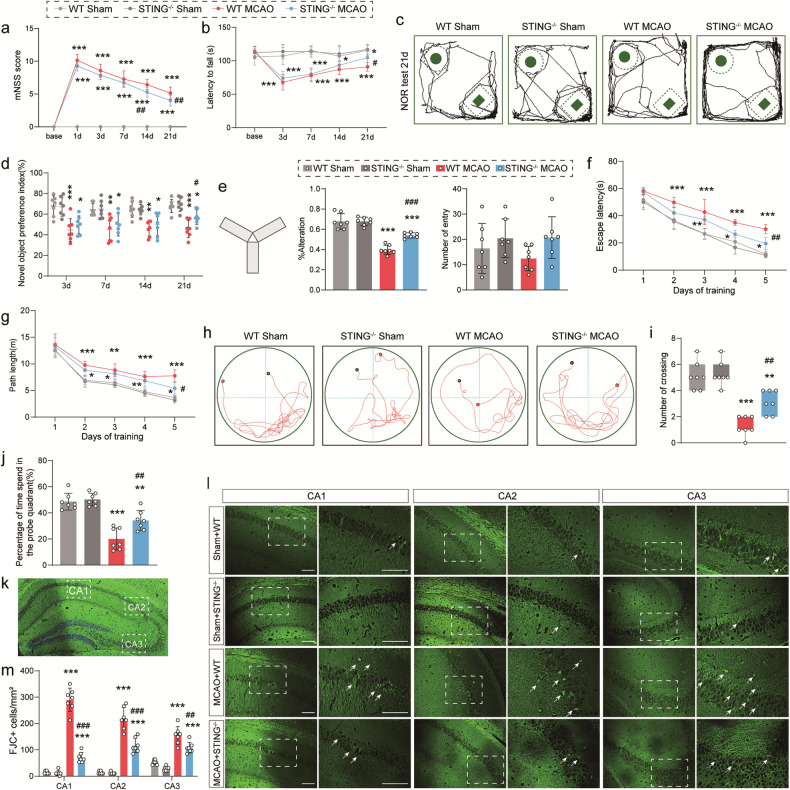
Ablation of STING rescued neurological deficits and cognitive dysfunction of MCAO mice. **a** Modified neurological severity score (mNSS) was used for sensorimotor function assessment after the MCAO surgery. **b** Rotarod assessment of WT and STING^−/−^ mice. STING^−/−^ mice performed significantly better than WT mice 21 days after ischemic stroke. **c** Novel object recognition (NOR) 21 days after MCAO or Sham operation. Representative movement paths shown as black lines. The dotted circles indicated the object exploration zones. Circle: familiar object. Rectangle: novel object. **d** The percentage of time exploring the novel object. **e** Y-maze test of WT and STING^−/−^ mice at 22 days after MCAO surgery. Bar graphs representing alteration percentage and number of entries. The escape latency (**f**) and path length (**g**) in the hidden platform phase. **h** Representative swimming paths of mice searching for the removed platform in the probe trial. **i**, **j** Time in the target quadrant and platform crossings were recorded in the probe trial. **k** Representative micrograph demonstrating the hippocampus area delineated by FJC staining. **l**, **m** FJC staining in the hippocampus regions results showed that the number of FJC-positive cells was increased 28 days after MCAO, which was decreased by STING knockout. Insets depict images at higher magnification. Data are expressed as mean ± SD. *n* = 7. Scale bar = 100 μm. ^*^P < 0.05, ^**^P < 0.01, ^***^P < 0.001 vs Sham mice. ^#^*P* < 0.05, ^##^*P* < 0.01, ^###^*P* < 0.001 vs WT MCAO mice.

Next, whether deleting STING impaired motion, learning and memory behavior in mice was checked. The mNSS results showed that MCAO mice demonstrated persistent sensorimotor deficits greatly reversed by STING-KO (Fig. 3a). Meanwhile, the rotarod and NOR tests were used for examining sensorimotor and memory functions before and up to 21 days post-MCAO surgery. STING ablation mice exhibited significantly strengthened motor coordination indicated by the increase of latency to fall off the rotarod (Fig. 3b), and improvement in memory function demonstrated by the increased novel object preference index in the NOR test (Fig. 3c, d) compared to WT ones at 21 days after reperfusion. To further investigate the long-term memory-related behavioral changes in the gene silencing of STING, Y-maze and MWM tests were performed 22 days after MCAO surgery. It was discovered that a decrease in alteration percentage of mice with the Y-maze test was restored by the genetic silencing of STING (Fig. 3e), which indicated the rescue of the impaired spatial long-term memory. In the learning phase of MWM, the latency and path length to the hidden platform of MCAO mice were decreased by the silencing of STING on the 4th to 5th day compared to that of WT mice (Fig. 3f, g). Moreover, STING-KO mice made more platform crossings and spent more time in the target quadrant compared with WT MCAO group after removing the platform during the probe trial (Fig. 3h–j). Additionally, WT and STING-KO mice in the Sham groups showed no difference after receiving the neurobehavior test.

Moreover, FJC specifically labels degenerating neurons in acute injuries [45]. As illustrated in Fig. 3k–m, FJC staining revealed that most FJC-positive cells exhibited irregular or small rounded profiles, lack of protrusions, indicative of widespread neuronal degeneration, and were easily distinguished from little non-specific staining based on morphology [46]. In STING-KO mice, the number of degenerating neurons was reduced by 75.4%, 46.5% and 30.4% in the CA1, CA2 and CA3 areas of the hippocampus, respectively, at 28 days after modeling. Together, these data suggested that STING deficiency ameliorated ischemia-induced motor coordination and cognitive learning defects in mice undergoing surgery.

### Skewness of ischemic‑induced pro‑ to anti‑inflammatory state in microglia by STING deficiency

**Fig. 4:**
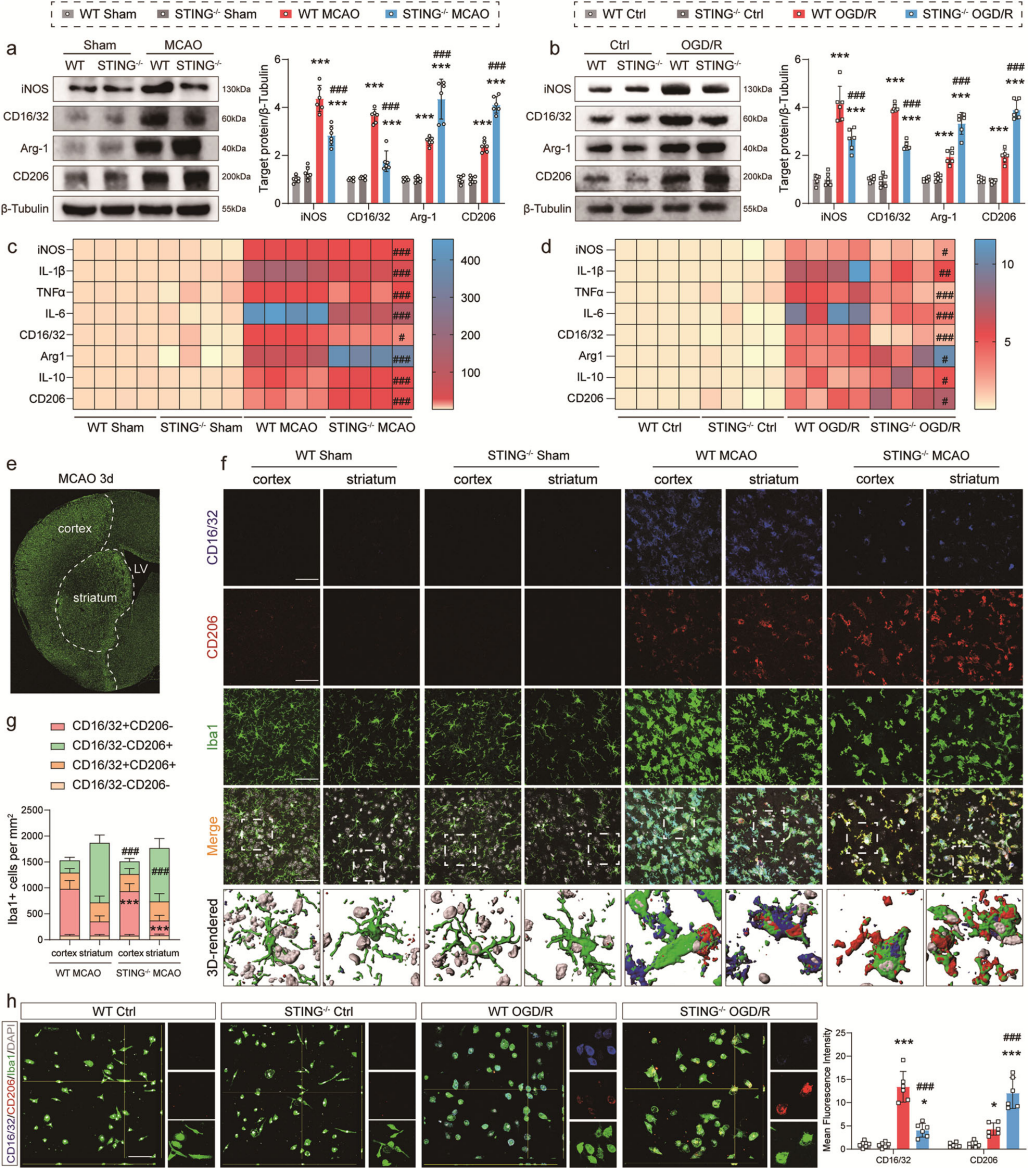
Effect of STING on microglial polarization in peri-infarct at 3 days post-MCAO and in primary microglial cells 24 h after OGD. **a**, **b** Representative western blot bands and quantifications of pro-inflammatory (CD16 and iNOS) and anti-inflammatory markers (CD206 and Arg-1). **c**, **d** qRT-PCR analyses of mRNA expressions of pro-inflammatory markers (iNOS, IL-1β, TNFα, IL-6 and CD16/32) and anti-inflammatory markers (Arg-1, IL-10 and CD206). *n* = 4. **e** Representative micrograph demonstrating the peri-infarct area delineated by Iba1 staining. **f**, **g** Representative photographs and 3D-reconstructed images of triple immunostaining of microglial polarization in the cortex and striatum of peri-infarct regions and quantitative analyses. **h** Immunofluorescence staining and 3D-reconstructed images of microglial polarization in primary microglia cells and quantitation of signals. Insets depict images at higher magnification. Pro-inflammatory phenotype: CD16/32^+^ (blue) and Iba1^+^ (green); anti-inflammatory phenotype: CD206^+^ (red) and Iba1^+^ (green). Data are expressed as mean ± SD. *n* = 6. Scale bar = 50 μm. ^*^P < 0.05, ^***^P < 0.001 vs Sham mice or Ctrl microglia. ^#^*P* < 0.05, ^##^*P* < 0.01, ^###^*P* < 0.001 vs WT MCAO mice or WT OGD/R microglia.

Based on previous research, STING is well characterized by its ability to augment local inflammation in ischemic brain [31]. It was proposed that the melt of STING might polarize microglia to anti-inflammatory state after reperfusion injury. First, primary STING-KO microglia were generated from STING-KO mice, and the western blot verified a complete lack of STING at the protein level (Figs. S4a, b). Then, the results of western blot revealed a statistical increase in the expression levels of pro-inflammatory microglia markers iNOS and CD16/32 and anti-inflammatory microglia markers Arg-1 and CD206 under ischemic stimulation in vivo or hypoxia stimulation in vitro (Fig. 4a, b). STING-KO decreased the pro-inflammation phenotype of microglia and increased the anti-inflammation phenotype, as evidenced by the declined expression of iNOS and CD16/32 and the elevated expression of Arg-1 and CD206 both in vivo or in vitro (Fig. 4a, b). The dramatic enhancement of Arg-1, interleukin-10 (IL-10), CD206 and other inflammatory cytokines was also detected in STING-KO mice by qRT-PCR in vivo and in vitro (Fig. 4c, d). Besides, STING-KO hindered the secretion of pro-inflammatory cytokines iNOS, IL-1β, TNFα, IL-6 and CD16/32 to a great extent (Fig. 4c, d). Next, triple-label immunofluorescent staining was performed for Iba1 and CD16/32 or CD206 3 days after MCAO surgery or 24 h after OGD/R treatment. WT mice and STING-KO mice in the Sham-operated group had few CD16/32^+^/Iba1^+^ or CD206^+^/Iba1^+^ cells in the cortex and striatum (Fig. 4e–g). However, STING-null MCAO mice showed a robust decrease in the number of CD16/32 and Iba1 co-stained cells and an increase in the number of CD206 and Iba1 co-stained cells in the cortex and striatum 3 days after reperfusion compared with WT MCAO ones (Fig. 4e–g). Consistently, it was found that the OGD/R group showed a dramatic increase in the number of anti-inflammatory phenotypes CD206^+^/Iba1^+^ in the administration of silence STING, but a significant decrease in the number of pro-inflammatory phenotypes CD16/32^+^/Iba1^+^ after OGD/R 24 h compared with the WT OGD/R group (Fig. 4h).

### Orchestration of infarct progression and microglial polarization phenotype by the specific re-expression of microglial STING

**Fig. 5:**
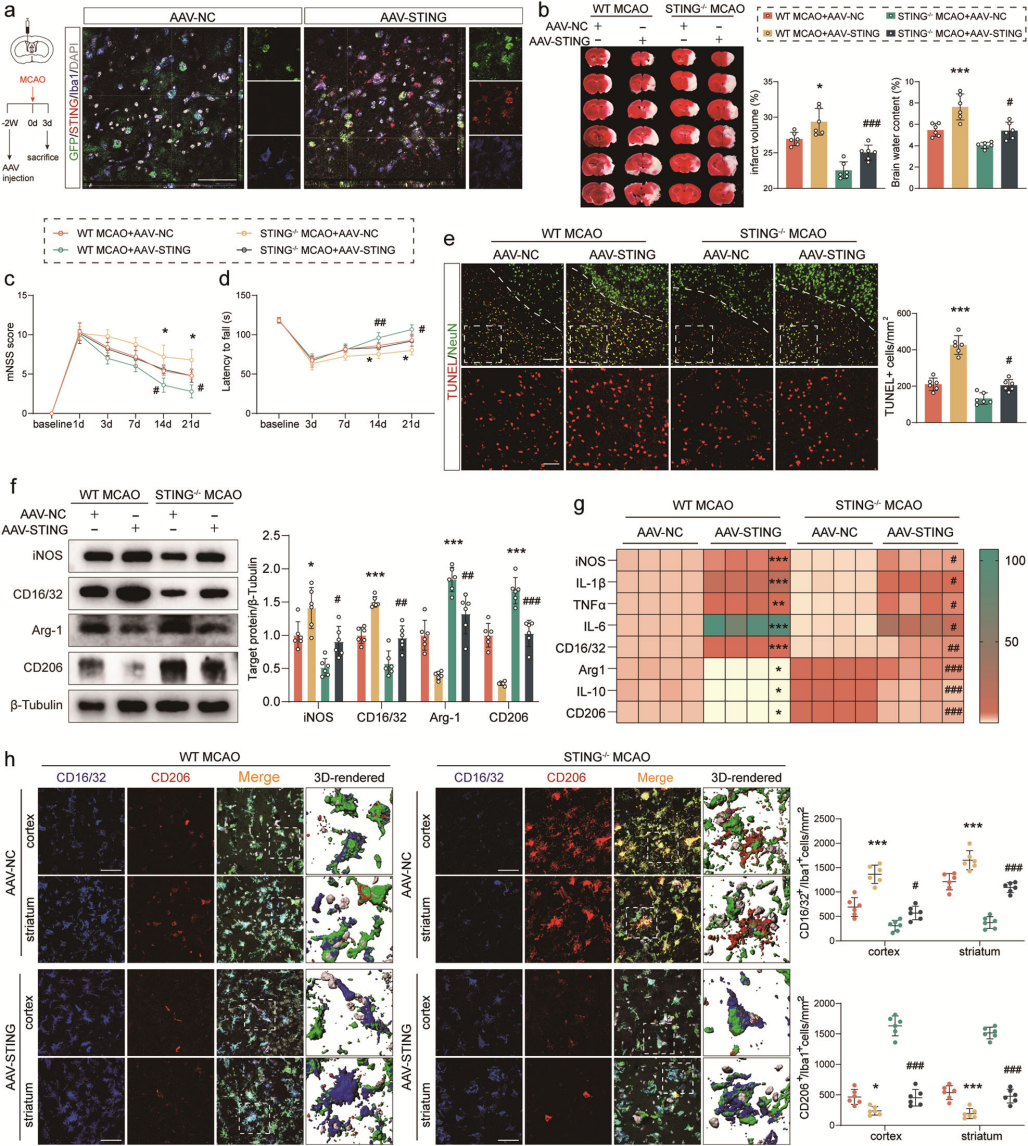
Specific STING upregulation in microglia aggravated infarct volume and promoted M1 microglia polarization after ischemic stroke. **a** AAV-NC or AAV-STING (green) co-stained with Iba1 (blue) and STING (red) 3 days post-MCAO in mice receiving EGFP-conjugated adeno-associated virus (AAV). **b** Representative TTC staining indicating ischemic infarct volume and brain edema in WT or STING^−/−^ MCAO mice injected with AAV-NC or AAV-STING. **c** The mNSS of WT MCAO, and STING^−/−^ MCAO mice injected with AAV-NC or AAV-STING. **d** Rotarod assessment of WT and STING^−/−^ mice receiving EGFP-conjugated AAV. Mice were tested before MCAO surgery. **e** TUNEL/NeuN staining in the peri-infarct area and quantitative analysis. Scale bar = 100 μm. **f** Immunoblotting image of microglial phenotype markers in WT or STING^−/−^ MCAO mice receiving AAV, with quantification. **g** qRT-PCR analyses of mRNA expressions of pro-inflammatory and anti-inflammatory factors in mice receiving AAV injection. **h** Representative photographs and quantitative analysis of microglial polarization at the cortex and striatum of the perilesional site by triple immunostaining. Data are expressed as mean ± SD. *n* = 6. Scale bar = 50 μm. ^*^P < 0.05, ^**^P < 0.01, ^***^P < 0.001 vs AAV-NC infected WT MCAO animals. ^#^*P* < 0.05, ^##^*P* < 0. 01, ^###^*P* < 0.001 vs AAV-NC infected STING^−/−^ MCAO animals.

A genetic approach was taken to specifically increase microglial STING using an AAV carrying the STING gene under the promoter of F4/80 (AAV-STING). Viral vectors were stereotaxically injected into the brains of mice 2 weeks before MCAO surgery (Fig. 5a). The EGFP signal was observed within Iba1^+^ microglia in STING-KO MCAO mice. AAV-STING successfully elevated the expression of microglial STING (Fig. 5a). TTC staining was applied to evaluate infarct volume 3 days after MCAO in AAV-STING-transfected mice. The results demonstrated that infarct volume and cerebral edema were increased dramatically after MCAO injury, escalating further in WT and STING-KO mice following STING gene transfection (Fig. 5b). Also, these changes were consistent with the development of neurological deficits and neuronal apoptosis. Sensorimotor coordination deficits aggravated in AAV-STING injected mice by the mNSS score and rotarod test compared with AAV-NC mice after MCAO (Fig. 5c, d). Concurrently, STING re-expression increased the number of apoptotic neurons (Fig. 5e). Besides, the intracerebroventricular injection of AAV-STING significantly increased the expression of pro-inflammatory factors (TNF-α, iNOS, IL-1β, IL-6 and CD16/32) and suppressed the expression of anti-inflammatory factors (Arg-1, IL-10 and CD206) both at protein and transcriptional levels compared with the null EGFP reporter virus according to western blot and qRT-PCR results (Fig. 5f, g). Similarly, the number of Iba1^+^CD16/32^+^ cells around the cortex and striatum of ischemic penumbra showed a noticeable increase in the MCAO mouse brain receiving AAV-STING (Fig. 5h). The cortex and striatum Iba1^+^CD206^+^ microglia were remarkably decreased in AAV-STING-transfected MCAO mice of both genotypes (Fig. 5h). These data suggested that STING was responsible for infarct progression and microglial polarization, which indicated that microglial STING may be an important intermediary in I/R injury.

### Regulation of microglial autophagy by STING both in vivo and in vitro

**Fig. 6:**
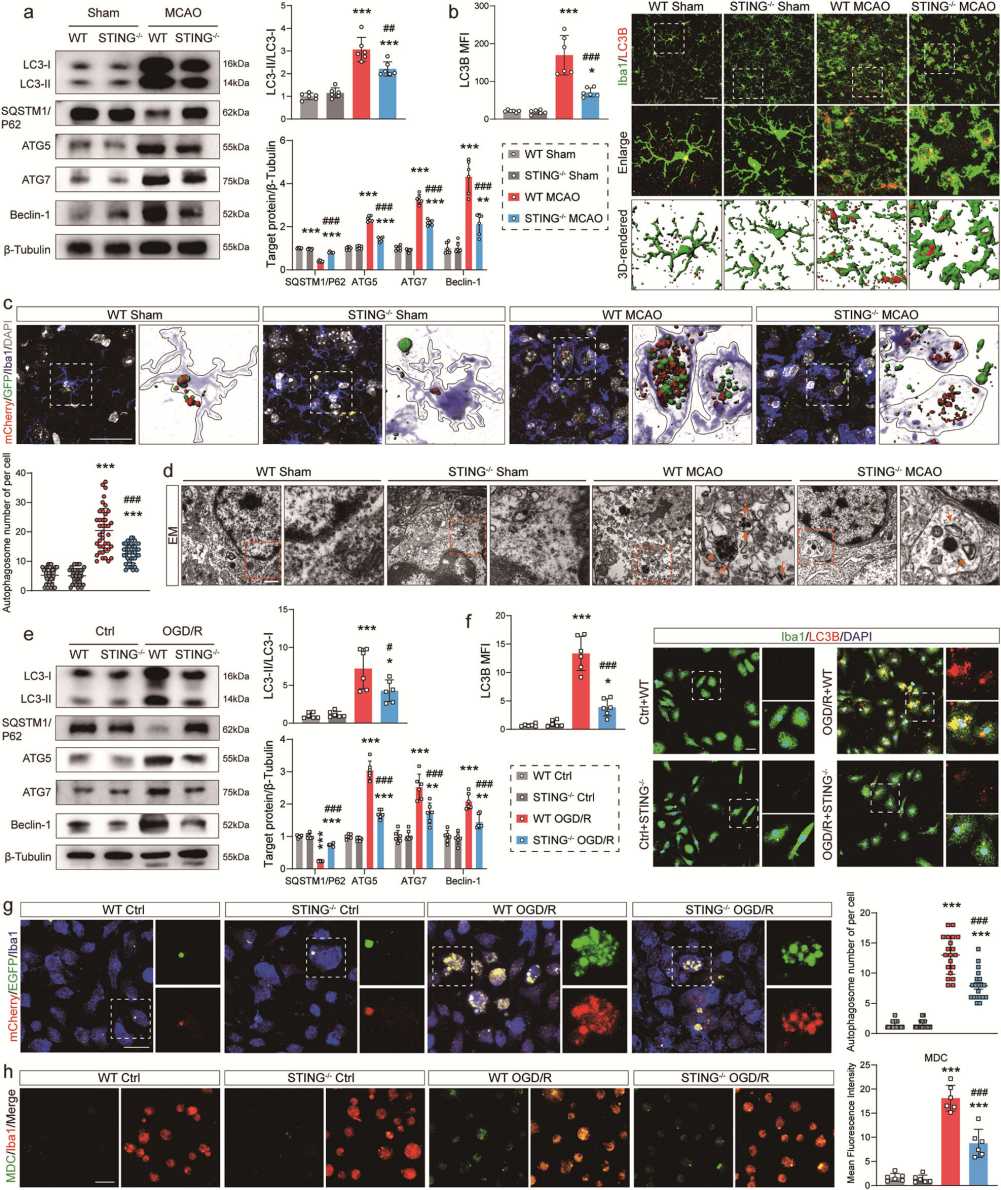
STING regulates I/R-induced autophagy in vivo and in vitro. **a** Representative immunoblots and quantification of LC3-I, LC3-II, SQSTM1/P62, ATG5, ATG7 and Beclin-1 in the ischemic penumbra from WT mice and STING^−/−^ mice 3 days after I/R. **b** Representative immunofluorescence images and mean fluorescence intensity quantification of Iba1/LC3B in the WT and STING^−/−^ mice. **c** Representative images showing mCherry-LC3 (red), GFP-LC3 (green), Iba1 (blue) and DAPI (gray, nuclei). Insets 3D-reconstructed images at higher magnification. Quantification of the number of yellow puncta (autophagosome). *n* = 20. **d** Representative transmission electron microscopy images of microglia in peri-infarct area. Magnified views of microglial autophagosome are marked with dashed line boxes. Orange arrow head: autophagosome. Scale bar = 500 nm. **e** Western blot analysis of LC3-I, LC3-II, SQSTM1/P62, ATG5, ATG7 and Beclin-1 in OGD primary microglia 24 h after reoxygenation. **f** Representative immunofluorescence images and mean fluorescence intensity quantification of LC3B taken from the primary microglia from WT mice and STING^−/−^ mice 24 h after OGD/R. **g** Representative images showing mCherry-LC3 (red), EGFP-LC3 (green) and Iba1 (blue). Quantification of the number of yellow puncta (autophagosome). *n* = 20. **h** Representative images of MDC (Monodansylcadaverine) staining for autophagosomes in cultured primary microglia. Data are expressed as mean ± SD. *n* = 6. Scale bar = 30 μm. ^*^P < 0.05, ^***^P < 0.01, ^**^P < 0.001 vs Sham mice or Ctrl microglia. ^#^*P* < 0.05, ^##^*P* < 0. 01, ^###^*P* < 0.001 vs WT MCAO mice or WT OGD/R microglia.

The changes of autophagy-associated proteins in the ischemic penumbra of WT and STING-KO mice were detected to determine the regulatory impact of STING on I/R-induced autophagy in vivo. It was noticed that MCAO surgery in WT mice contributed to a significant increase in the levels of LC3 II, ATG5, ATG7 and Beclin-1 and a decline in the level of SQSTM1/P62 3 days after I/R. However, STING deficiency markedly suppressed the levels of LC3 II, ATG5, ATG7 and Beclin-1 but increase the level of SQSTM1/P62 3 days after I/R. STING deficiency did not affect the levels of LC3 II, SQSTM1/P62, ATG5, ATG7 and Beclin-1 under non-ischemic conditions (Sham-operated groups) (Fig. 6a). Compared with Sham mice, WT MCAO ones had more numbers of LC3B-positive puncta in microglia 3 days after I/R. By comparison, a marked decrease in numbers of LC3B positive puncta occurred in STING-KO microglia (Fig. 6b). Autophagic flux was also examined by transfecting AAV-mCherry-GFP-LC3B to mice with or without STING. The results showed that the number of autophagosome puncta decreased in STING-KO mice with MCAO surgery (Fig. 6c), which indicated that autophagy was inhibited. Moreover, transmission electron microscopy demonstrated that autophagic vesicles and autophagosomes exhibited a rise in microglia after MCAO, but a reduction in STING-KO mice (Fig. 6d). After that, the expression of autophagy-associated markers in the primary microglia treated with OGD/R was examined in vitro. As illustrated in Fig. 6e, LC3 II, ATG5, ATG7 and Beclin-1 were all significantly increased, while SQSTM1/P62 was decreased at the protein level 24 h after the OGD/R insult. Immunofluorescence staining confirmed that the mean fluorescence intensity of microglial LC3B was reduced by STING-KO (Fig. 6f). Similarly, the number of autophagosome puncta exhibited a significant elevation in the WT OGD/R group with transfected Lenti-mCherry-EGFP-LC3B. STING-KO suppressed the increase of OGD/R-induced autophagic flux and decreased the number of autophagosome puncta (Fig. 6g). MDC dye specifically accumulating in autophagic vacuoles and autophagosome-containing cells was also used [43]. In line with other results, STING-KO weakened the mean fluorescence intensity of the MDC signal (Fig. 6h). These results indicate the regulation of I/R-induced autophagy by STING in ischemic stroke models.

### Skewness of STING-KO‑induced anti‑ to pro-inflammatory state in microglia due to irritating autophagy by rapamycin

**Fig. 7:**
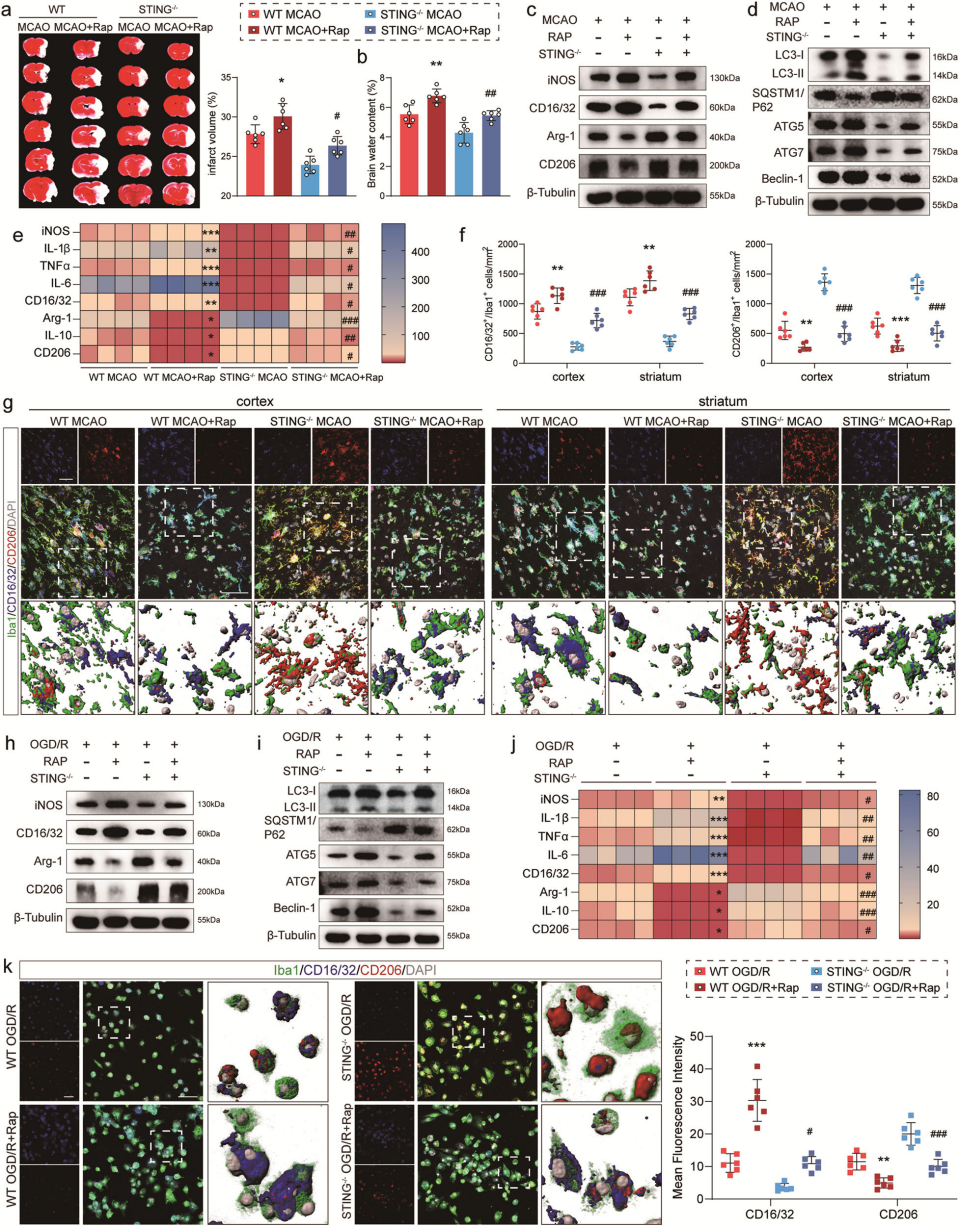
Autophagy activating aggravated the detrimental effects of STING after MCAO attack. **a**, **b** Representative micrographs depicting TTC staining and quantitation of infarct volume and brain edema following injected with rapamycin. **c** Immunoblot of iNOS, CD16/32, Arg-1, and CD206 in mice after injected with rapamycin. **d** The expression of autophagy-related proteins (LC3-I, LC3-II, SQSTM1/P62, ATG5, ATG7 and Beclin-1) after rapamycin injected were detected by western blot. **e** Relative mRNA expression of pro-inflammatory specific genes and anti-inflammatory specific genes after rapamycin injected. **f**, **g** Triple immunostaining of Iba1, CD16/32 and CD206 in the cortex and striatum of peri-infarct regions and quantitative analysis 3 days following rapamycin injected. **h** Representative western blot bands of iNOS, CD16/32, Arg-1, and CD206 in cultured primary microglia incubated with rapamycin. **i** The expression of autophagy-related proteins in cultured microglia after incubated with rapamycin. **j** qRT-PCR analyses of mRNA expressions of pro-inflammatory markers (iNOS, IL-1β, TNFα, IL-6 and CD16/32) and anti-inflammatory markers (Arg-1, IL-10 and CD206) in cultured primary microglia. *n* = 4. **k** Representative immunostained images and statistical analysis of microglial marker Iba1 with CD16/32 or CD206 in primary microglia after co-cultured with rapamycin. Data are expressed as mean ± SD. *n* = 6. Scale bar = 30 μm. ^*^P < 0.05, ^**^P < 0.01, ^***^P < 0.001 vs WT MCAO mice or WT OGD/R microglia. ^#^*P* < 0.05, ^##^*P* < 0. 01, ^###^*P* < 0.001 vs STING^−/−^ MCAO mice or STING^−/−^ OGD/R microglia.

To evaluate the effect of autophagy activation on the transition of microglia phenotypes, autophagy inducer rapamycin was used to aggravate autophagy and the changes in infarction volumes in mice were observed. Activation autophagy with rapamycin aggravated the baleful effects of I/R injury on brain infarction and edema in the MCAO mice of both genotypes (Fig. 7a, b). It was noted that the administration of rapamycin resulted in a dramatic increase in the expression of iNOS and CD16/32, two pro-inflammatory phenotype markers, compared to the WT MCAO group. Furthermore, a marked decrease occurred in the expression of Arg-1 and CD206, two anti-inflammatory phenotype markers. This pro-inflammatory effect was decreased in STING-KO mice post-MCAO but was enhanced when STING-null mice were treated with rapamycin (Figs. 7c and S5a). In addition, the same trend was seen in primary microglia experiments (Figs. 7h and S5d). Moreover, the western blot results showed that rapamycin treatment in vivo and in vitro elevated the levels of LC3, ATG5, ATG7 and Beclin-1 but decreased the level of SQSTM1/P62 (Figs. 7d, i, S5b, c, e, f). The qRT-PCR analysis demonstrated that the increased levels of pro-inflammatory cytokines (iNOS, IL-1β, TNF-α, IL-6 and CD16/32) and the mitigated levels of anti-inflammatory factors (Arg-1, IL-10 and CD206) in WT and STING-KO mice after rapamycin treatment (Fig. 7e). In line with observation in vivo, rapamycin incubation in vitro also resulted in a significant shift towards a pro-inflammatory functional status in both WT and STING-KO microglia (Fig. 7j). Additionally, CD16/32^+^Iba1^+^ cells increased in number after rapamycin treatment compared to the WT MCAO group. Simultaneously, rapamycin significantly decreased the number of CD206^+^Iba1^+^ cells after MCAO compared with no-treatment group (Fig. 7f, g). As depicted in Fig. 7k, the mean fluorescence intensity of CD16/32 exhibited a significant increase after treatment with rapamycin, while that of CD206 demonstrated an obvious decrease. Based on the research, it was reasonable to conclude that autophagy was important in the transformation of microglia phenotypes.

### Orchestration of autophagy by STING through direct interaction with LC3 via the TM domain after the I/R insult

**Fig. 8:**
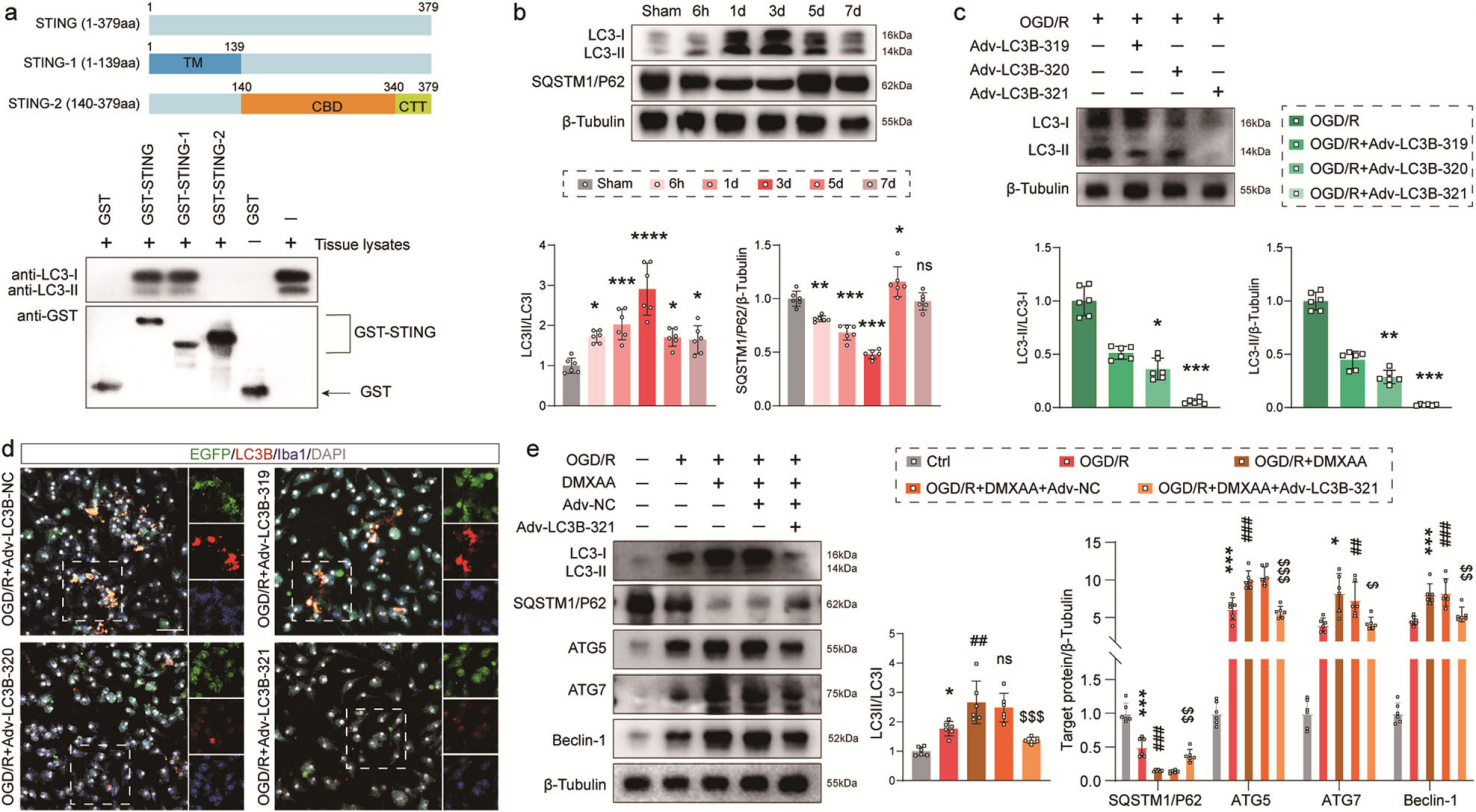
LC3 was involved in STING-induced microglial polarization on I/R injury. **a** Schematic representation of STING fusion proteins. Interaction was detected between STING domain (1-139aa) and LC3-II. **b** Representative immunoblots and quantification of LC3-I, LC3-II and SQSTM1/P62 in ischemic brain at the indicated time points after I/R. *n* = 6. ^*^P < 0.05, ^**^P < 0.01, ^***^P < 0.001 vs Sham mice. ns., no significant difference. **c** Immunoblots and quantitative analysis of Adv-LC3B-319, Adv-LC3B-320 and Adv-LC3B-321. *n* = 6. ^*^P < 0.05, ^**^P < 0.01, ^***^P < 0.001 vs OGD/R + Adv-LC3B-319 group. **d** Transfection efficiency of Adv-NC, Adv-LC3B-319, Adv-LC3B-320 and Adv-LC3B-321 in cultured primary microglia. Scale bar = 50 μm. **e** Western blot analysis and quantification of autophagy-related proteins in cultured primary microglia. *n* = 6. ^*^P < 0.05, ^***^P < 0.001 vs OGD/R group. ^##^*P* < 0. 01, ^###^*P* < 0.001 vs OGD/R + DMXAA group. ^$^*P* < 0.05, ^$$^*P* < 0. 01, ^$$$^*P* < 0.001 vs OGD/R + DMXAA + Adv-NC group. ns., no significant difference. Data are expressed as mean ± SD.

Recently, the induction of autophagy via STING trafficking has been reported to be a fundamental function of the cGAS-STING pathway [26]. The immunostaining results showed that STING co-localized with LC3 in Iba1^+^ microglia after stroke. Besides, ERGIC with STING is a membrane source for the lipidation of LC3, a crucial step in the biogenesis of autophagosomes [26]. As a result, the focus was placed on the LC3 protein of the autophagy pathway for further investigation into STING-involved autophagy after I/R injury in the brain. STING possesses multiple functional domains that can combine with a variety of signaling proteins. The domain of STING binding to LC3 was determined by synthesizing and purifying two GST-tagged STING domains (1-139aa and 140-379aa, Fig. 8a), and incubating them with the tissue lysates of cerebral ischemic penumbra. The particular protein-to-protein interaction was observed between the STING domain (1-139aa) and LC3 (Fig. 8a). The protein-protein docking simulation predicted the structure of STING domain (1-139aa) and LC3 interaction (Fig. S6). To probe into the dynamic changes of LC3 and SQSTM1/P62 expression, western blot was performed for the assessment of penumbral area samples from Sham and MCAO mice at 6 h, 1, 3, 5 and 7 days after modeling. The western blot results signified that LC3 showed a marginal increase at 6 h after surgery, reached the peak at 3 days after reperfusion, and exhibited a gradual decline. The protein level of SQSTM1/P62 presented a continual reduction from 6 h to 3 days after I/R and then a gradual rebound at 5 and 7 days after reperfusion (Fig. 8b). Given the upstream regulation of STING on LC3 in autophagy, whether the suppression of LC3 could weaken STING-induced detrimental effects after ischemia was questioned. Adv strategy was adopted to suppress the expression of LC3 in microglia. Compared with Adv-LC3B-319 and Adv-LC3B-320, Adv-LC3B-321 transfection inhibited the expression of the LC3 protein in OGD/R microglial cells (Fig. 8c). Immunofluorescence images revealed that Adv-LC3B-321 transfection down-regulated the expression of the microglial LC3 protein under OGD/R stress (Fig. 8d). Hence, Adv-LC3B-321 or Adv-NC used for subsequent assays was utilized to transfect cells. Then, an agonist called DMXAA was introduced to augment the expression of STING. As shown in Fig. 8e, the expression of autophagy-related proteins was exacerbated under the administration of DMXAA and the transfection of Adv-NC, which were ameliorated by Adv-LC3B-321 administration. These findings indicate that microglia STING could be pivotal in regulating autophagy processes after ischemia by directly interacting with LC3 via the transmembrane (TM) domain.

## Discussion

**Fig. 9:**
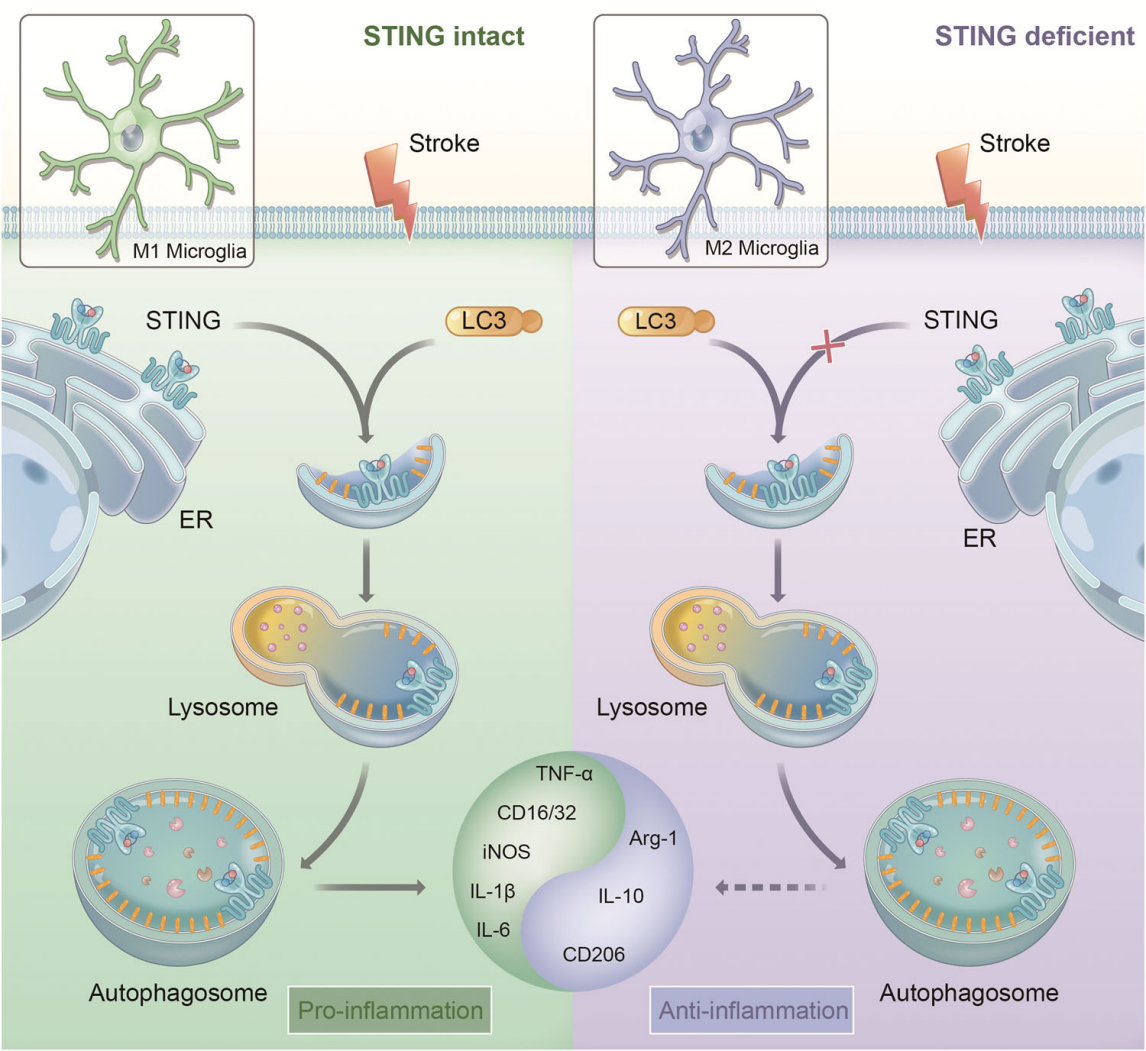
Schematic diagram of STING-mediated autophagy-driven microglial polarization following ischemic stroke. Brain ischemia induces STING activation, enhancing STING-LC3 interaction and autophagy, driving the shift of microglia polarization phenotype towards a pro-inflammatory direction. Knockout of STING shifts microglia towards an anti-inflammatory modality by inhibiting autophagy, thereby rescuing neuroinflammation and improving stroke outcomes.

Neuroinflammation is recognized as a key driver of secondary injury progression after ischemic stroke. Microglia, known as the first immune cells in the CNS to reach the injured area, constitute a major component of neuroinflammation development after ischemic stroke [47, 48]. Their functions can be either neurotoxic and neuroprotective, responding to different stimuli to acquire distinct phenotypes and functions [10, 49]. Thus, it is of particular importance to subtly adjust the balance between microglia phenotypes. Our previous study showed that STING was able to aggravate post-ischemic stroke neuroinflammation by mediating the polarization of microglia to M1 phenotype [31]. However, its downstream factors and specific mechanisms remain unclear. Here, it was shown that STING activated autophagy through direct binding to LC3 via its TM domain, which drove microglia towards pro-inflammatory phenotype, intensifying post-stroke neuroinflammation. These inflammatory reactions were accompanied by an increase in infarct volume, neuronal injury as well as long-term functional impairment. STING-KO or the suppression of LC3B expression by Adv strategy switched microglia towards anti-inflammatory phenotype and improved long-term neurobehavioral function (Fig. 9). Consequently, modulating microglial autophagy may represent a novel strategy for the regulation of inflammatory environment.

cGAS-STING signaling triggers innate immune response by sensing cytosolic DNA and is found to be activated in most CNS diseases [25, 50, 51]. The excessive activation of this pathway contributes to microglia-mediated neuroinflammation and nerve cell death [31, 44, 52]. For instance, Gamdzyk and his colleagues demonstrated that activation of STING through exogenous 2’3’-cGAMP could promote neuronal death and reverse the therapeutic effect of cGAS inhibitor after hypoxic ischemic encephalopathy [53]. Peng et al. reported that pharmacologically inhibiting STING could attenuate inflammatory injury partly by activating AMPK signal in SAH [30]. STING deletion suppressed NLRP3-drived microglial pyroptosis, which was accompanied by the mitigation of neuronal damage and neurobehavioral impairment [44]. However, these studies generally employ specific antagonists or siRNA to alter the function of STING, and STING-KO mice are required for the further investigation of ischemic stroke. Extending on prior research with STING inhibitors C176 and siRNA, we genetically removed STING in mice to study its effect on microglial polarization post-stroke. It was observed that the absence of STING gene changed the direction of microglial polarization.

Macrophages/microglia polarization is critical element of the innate immune response, characterized by the distinct secretion profiles of inflammatory mediators. Inflammatory mediators are typically orchestrated by innate immune pathways, especially the cGAS-STING axis. Previously, it was reported that cGAS-STING signaling cascade affected macrophages polarization in cancer, autoimmune and inflammatory diseases. The deficiency of LC3-associated phagocytosis induces a STING-mediated IFN-I response in tumor-associated macrophages (TAMs), driving TAMs toward an M1 phenotype associated with anti-tumor immunity [54]. Nevertheless, contradictory evidence illustrates that during Streptococcus pyogenes infection, STING supports IFN-I -induced M2 macrophage polarization, dampening inflammatory response to infection [55]. Additionally, the function of cGAS should not be overlooked. Sensing DNA from myocardial cells damaged by ischemia triggers cGAS, affecting macrophage polarization. cGAS loss-of-function favor a reparative macrophage phenotype, thereby augmenting myocardial repair [56]. Notably, cGAS induced macrophage polarization to the M1 phenotype via the mTORC1 pathway under LPS stimulation, seemingly independent of STING [57]. Regarding CNS injury, neuronal endoplasmic reticulum (ER) stress activates STING signaling, triggering IFN-β release, which increase microglial M1 polarization, worsening traumatic brain injury [58]. Our previous work has revealed that the IRF3/NF-κB pathway is involved in the modulation of microglial polarization by STING following ischemic stroke [31]. Overall, cGAS-STING activation sets off immune cascades, driving macrophage polarization. Beyond this, this study uncovered the involvement of autophagy in the regulation of microglial polarization post-stroke.

Recent studies have shown the importance of autophagy in ischemic stroke, where autophagy engages in a complex crosstalk with inflammation [18, 59]. On the one hand, autophagy is thought to inhibit inflammation under some circumstances. In Parkinson’s disease, it partly steer microglial polarization towards M2 phenotype through the AKT/mTOR pathway, thereby curbing inflammation [60]. In addition, another investigation has declared that ischemic brain damage was aggravated by the administration of autophagic inhibitor 3-methyladenine (3-MA) [61]. On the other hand, there are conflicting opinions that suppression of autophagy decreased microglia activation and inflammatory injury to neurons through TLR4, thereby ameliorating the neurological symptoms of ICH mice [21]. This finding was similarly corroborated by Zhang et al., who indicates that 3-MA partially avert neuronal injury induced by enhancement of autophagy after I/R injury [62]. This duality highlights the nuanced role of autophagy in inflammation, which likely depends on the specific disease context and the stage of the inflammatory process. In our study, autophagy markers LC3 and SQSTM1/p62 exhibited time-dependent expression changes in the ischemia-reperfusion model, indicating that the varying manifestations of autophagy may be closely related to the duration of cerebral I/R. Besides, our study used male-only model and primary microglia to validate that STING influences microglial polarization via autophagy, minimizing variables related to sex differences. However, autophagy signaling may be sex-related, possibly through sex hormones and their receptors [63]. Future investigations should include female models to explore sex-specific regulatory mechanisms.

Researchers have always paid attention to the effect of autophagy on the phenotypic transformation of microglia. The mainstream view holds that autophagy can regulate microglial polarization by affecting its secretion of inflammatory factors [20, 60]. It was found that autophagy promoted the secretion of numerous pro-inflammatory factors by microglia and aggravated its pro-inflammation phenotype after stroke. But, some studies also discovered that autophagy inhibits the polarization of M1-Type microglia by clearing reactive oxygen species [64]. Furthermore, it has been proposed that autophagy is involved in the renewal of organelles like mitochondrial autophagy, which may affect function of microglia polarization state through metabolism distortion [65]. These mechanisms suggest that autophagy plays a vital part in the appearance and progression of neurological diseases by influencing microglia polarization through a variety of pathways, but specific molecular mechanisms need further exploration.

Another focus of this study was to investigate the potential molecular mechanism by which STING regulates autophagy. As reported by Gui et al., STING could activate autophagy independent of canonical TANK-binding kinase 1 (TBK1) activation and interferon induction [26]. Liu et al. claimed that STING directly interacts with LC3 through its conserved LC-3 interacting regions (LIR) motifs during herpes simplex virus Type 1 (HSV-1) infection to induce autophagy [66]. Likewise, our findings show that STING induces autophagy through the interaction of its TM domain with LC3. This interaction is critical for the formation of autophagosomes. Moreover, STING migrates from ER to ERGIC and Golgi, in a process governed by the COP-II complex and ARF GTPases [67]. The vesicles budding from the ER and ERGIC could serve as membrane sources for LC3 lipidation and autophagosome biogenesis [68]. As highlighted, this process bypasses upstream autophagy regulators, but depends on downstream autophagy regulators such as Atg5 and Atg16L1 [69]. These studies suggest that STING initiates non-canonical autophagy by interacting with LC3. Further, alternative viewpoints exist concerning the mechanism of STING in relation to autophagy. Autophagy-adaptor protein P62 can interact with STING and negatively controls the cGAS-STING pathway in stimulated cells [70], implying a regulatory feedback loop. Other researchers believe that the regulation of the autophagy process by STING is achieved via the direct interaction between STING and WIPI2 to induce the formation of autophagosomes [71]. In addition, STING may regulate autophagy by influencing energy metabolism, particularly through its interaction with Syntaxin 17 [72]. Meanwhile, STING-KO can accelerate the fusion process of autophagosomes and lysosomes, relying on AMPK regulatory proteins in the classical autophagy pathway-in response to energy stress. Moreover, STING orchestrates ER-phagy, a specialized form of autophagy critical for handling ER stress and maintaining ER homeostasis [73]. Despite these insights, the precise molecular details of how STING tunes autophagy, particularly under cerebral ischemic conditions, remain to be fully elucidated.

A limitation of our study is the exclusive use of Iba1 to label microglia. Although Iba1 is a widely used marker for microglia, it non-specifically labels other macrophage populations and does not encompass all microglia. We could partly exclude other Iba1^+^ CNS macrophages based on their distinct morphology and location. Thus, further rigorous study is warranted.

To sum up, the data of this study indicated the vital role of STING in neuroinflammation after cerebral ischemia. It was shown that microglial STING orchestrated autophagy via direct interaction with LC3 and caused the skewness of ischemia-activated microglia to a pro-inflammation state, which subsequently led to the production of inflammatory cytokines and neuroinflammation. In this study, the unrecognized function of the STING-LC3 axis in promoting microglial polarization was identified. Altogether, this work provides a possible treatment target for treating ischemic stroke, which opens up new possibilities for enhancing neuroprotection after stroke injury.

## Supplementary information

Supplementary Figure Legends

Supplementary Figure 1

Supplementary Figure 2

Supplementary Figure 3

Supplementary Figure 4

Supplementary Figure 5

Supplementary Figure 6

Supplementary table

Supplementary materials-original bolts
