# Supplementary material for: STING orchestrates microglia polarization via interaction with LC3 in autophagy after ischemia
Source: Cell Death Dis. 2024 Nov 13;15(11):824. doi: 10.1038/s41419-024-07208-1 (PMC11560960; doi:10.1038/s41419-024-07208-1)
Supplement: Supplementary file 1 — Supplementary Figure Legends [file 41419_2024_7208_MOESM1_ESM.docx]

**Supplementary Figure Legends**

**Supplementary Fig. 1 Experimental design and animal groups.**

STING, Stimulator of IFN genes; MCAO, middle cerebral artery occlusion; TTC, 2,3,5-triphenyl tetrazolium chloride solution; NOR test, novel object recognition test; qRT-PCR, quantitative reverse transcription polymerase chain reaction; i.c.v, intracerebroventricular injection; AAV, adeno-associated virus; NC, negative control.

**Supplementary Fig. 2 Occlusion of MCA decreased cerebral blood flow (CBF).**

Representative images and quantitative analyses showed the CBF before ischemia, during ischemia, 15 min after reperfusion and 3 d after reperfusion. Data are expressed as mean ± SD. n = 6. ^***^*P* < 0.001 vs Baseline group. ^###^*P* < 0.001 vs Ischemia group.

**Supplementary Fig. 3 The body weight and rotarod test.**

(a) The body weight showed STING^–/–^ mice were developed normally as WT mice. (b) The rotarod test showed no significant differences in motor coordination and locomotor activity between WT and STING^–/–^ mice. Data are expressed as mean ± SD. n = 30. ns., no significant difference.

**Supplementary Fig. 4 STING was completely knocked out at the protein level in** **cultured primary microglia from STING^–/–^ mice.**

Immunoblot assessment of STING and p-STING (a) and quantitation (b) in cultured primary microglia. Data are expressed as mean ± SD. n = 6. ^*^*P* < 0.05 vs WT Ctrl group. ^###^*P* < 0.001 vs WT OGD/R group.

**Supplementary Fig. 5 Rapamycin reinforces autophagy effects and regulates microglial polarization in vivo and in vitro.**

(a) Immunoblots quantification on the expression of iNOS, CD16/32, Arg-1, and CD206 in mice brain after injected with rapamycin. (b, c) Immunoblots quantification of LC3-I, LC3-II, SQSTM1/P62, ATG5, ATG7 and Beclin-1 after MCAO injury on WT mice and STING^–/–^ mice injected with rapamycin. (d) Quantification of iNOS, CD16/32, Arg-1, and CD206 protein expression under OGD/R stress in primary microglia treated with rapamycin. (e, f) Western blot analysis of autophagy-related proteins in primary microglia under OGD/R stress with or without rapamycin incubation. Data are expressed as mean ± SD. n = 6. ^**^*P* < 0.01, ^***^*P* < 0.001 vs WT MCAO mice or WT OGD/R microglia. ^##^*P* < 0.01, ^###^*P* < 0.001 vs STING^–/–^ MCAO mice or STING^–/–^ OGD/R microglia.

**Supplementary Fig. 6 Outline of molecular docking simulation performed for STING-LC3 interaction.**

Interaction analysis between STING and LC3 Proteins. Red sticks represent amino acid residues of STING protein, while cyan sticks depict those of LC3 protein. Green dashed lines indicate hydrogen bonds, orange dashed lines represent salt bridges, and red dashed lines show electrostatic interactions.
