# Supplementary figures and images for: STING orchestrates microglia polarization via interaction with LC3 in autophagy after ischemia
Source: Cell Death Dis. 2024 Nov 13;15(11):824. doi: 10.1038/s41419-024-07208-1 (PMC11560960; doi:10.1038/s41419-024-07208-1)

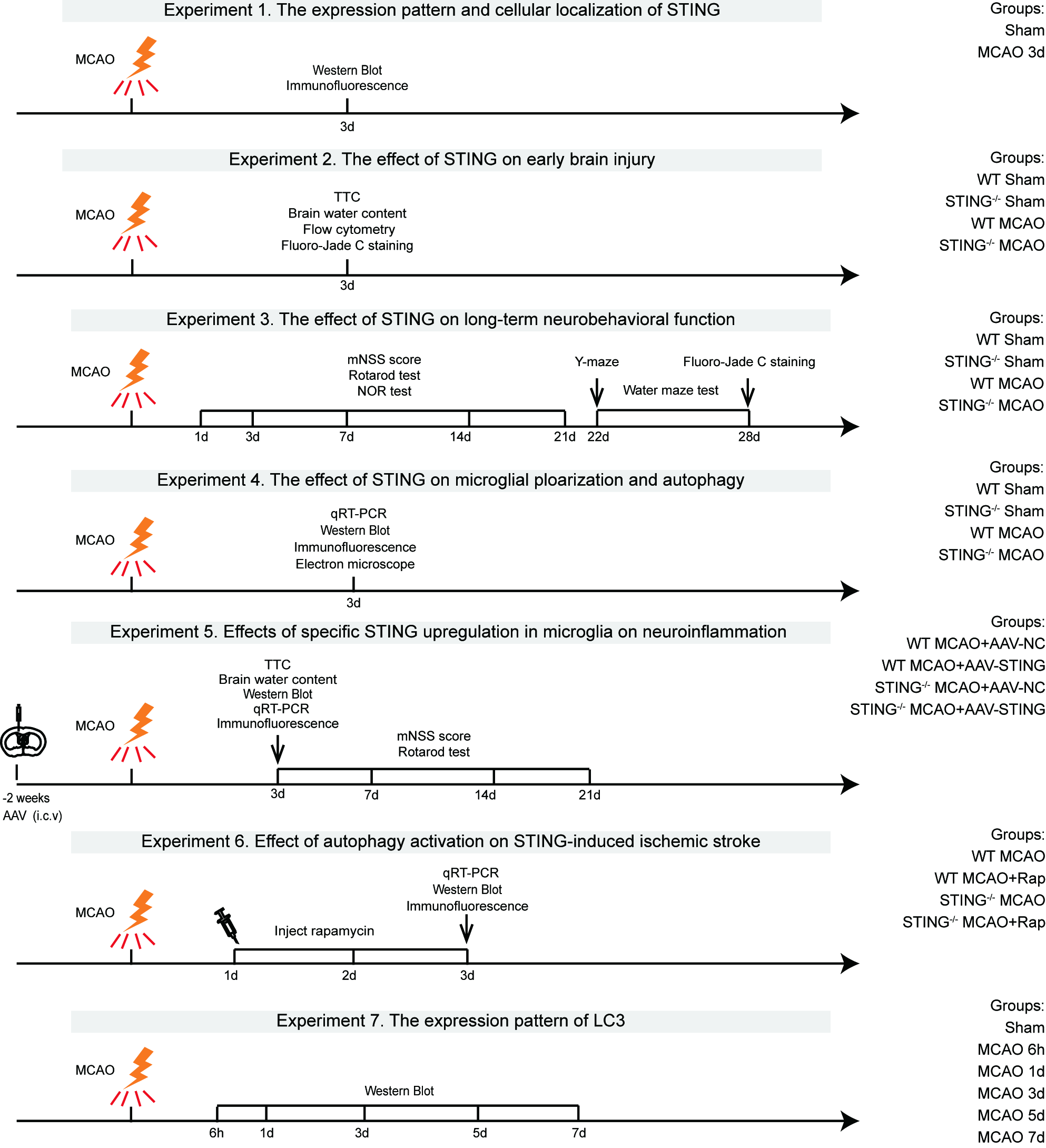

Supplement: Supplementary file 2 — Supplementary Figure 1 [file 41419_2024_7208_MOESM2_ESM.tif]

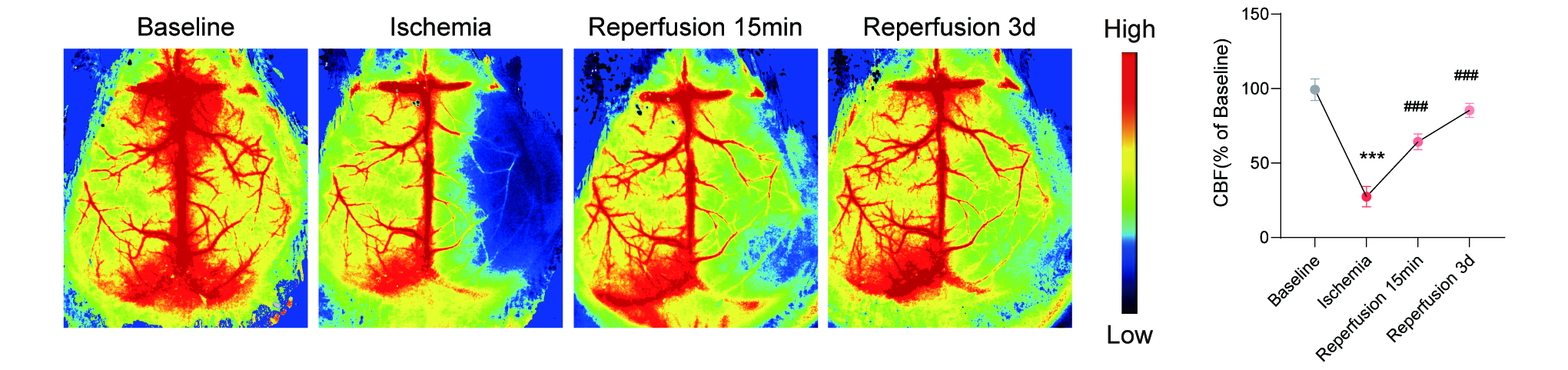

Supplement: Supplementary file 3 — Supplementary Figure 2 [file 41419_2024_7208_MOESM3_ESM.tif]

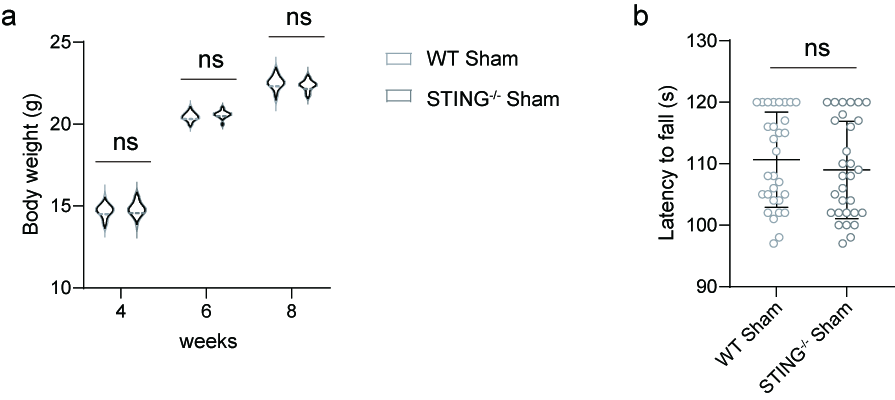

Supplement: Supplementary file 4 — Supplementary Figure 3 [file 41419_2024_7208_MOESM4_ESM.tif]

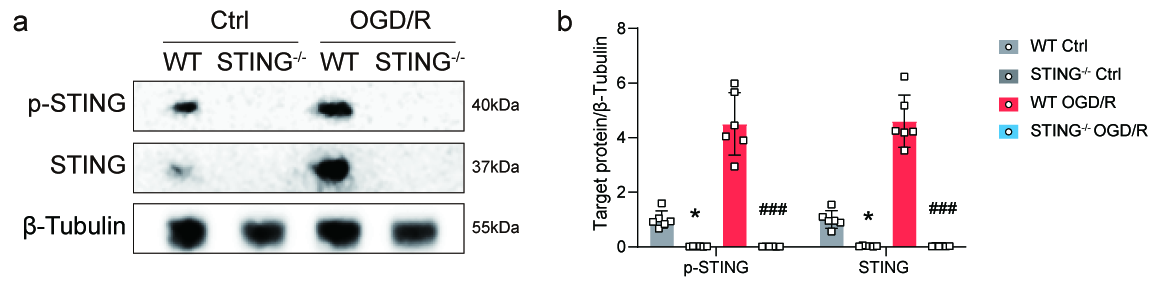

Supplement: Supplementary file 5 — Supplementary Figure 4 [file 41419_2024_7208_MOESM5_ESM.tif]

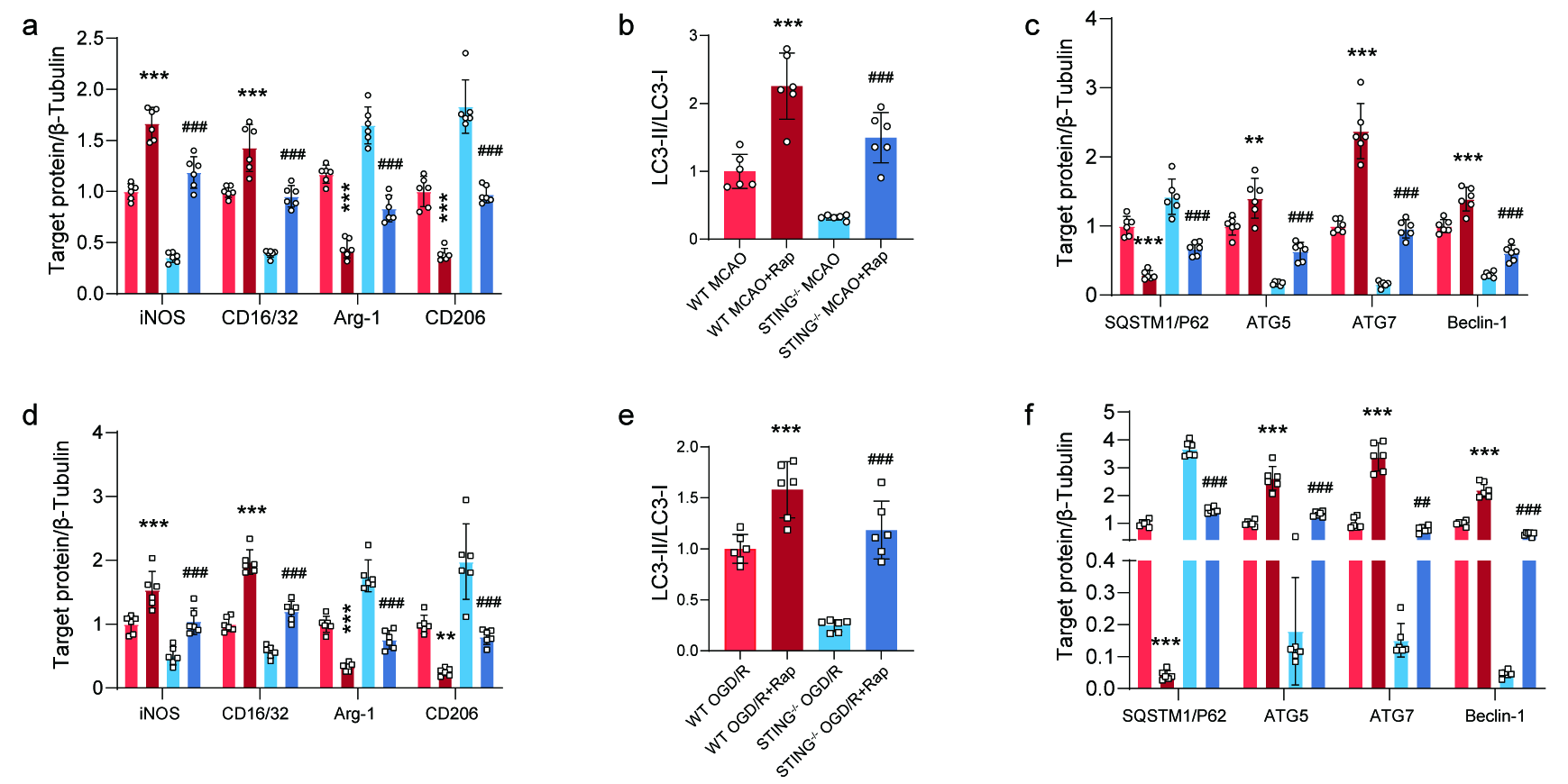

Supplement: Supplementary file 6 — Supplementary Figure 5 [file 41419_2024_7208_MOESM6_ESM.tif]

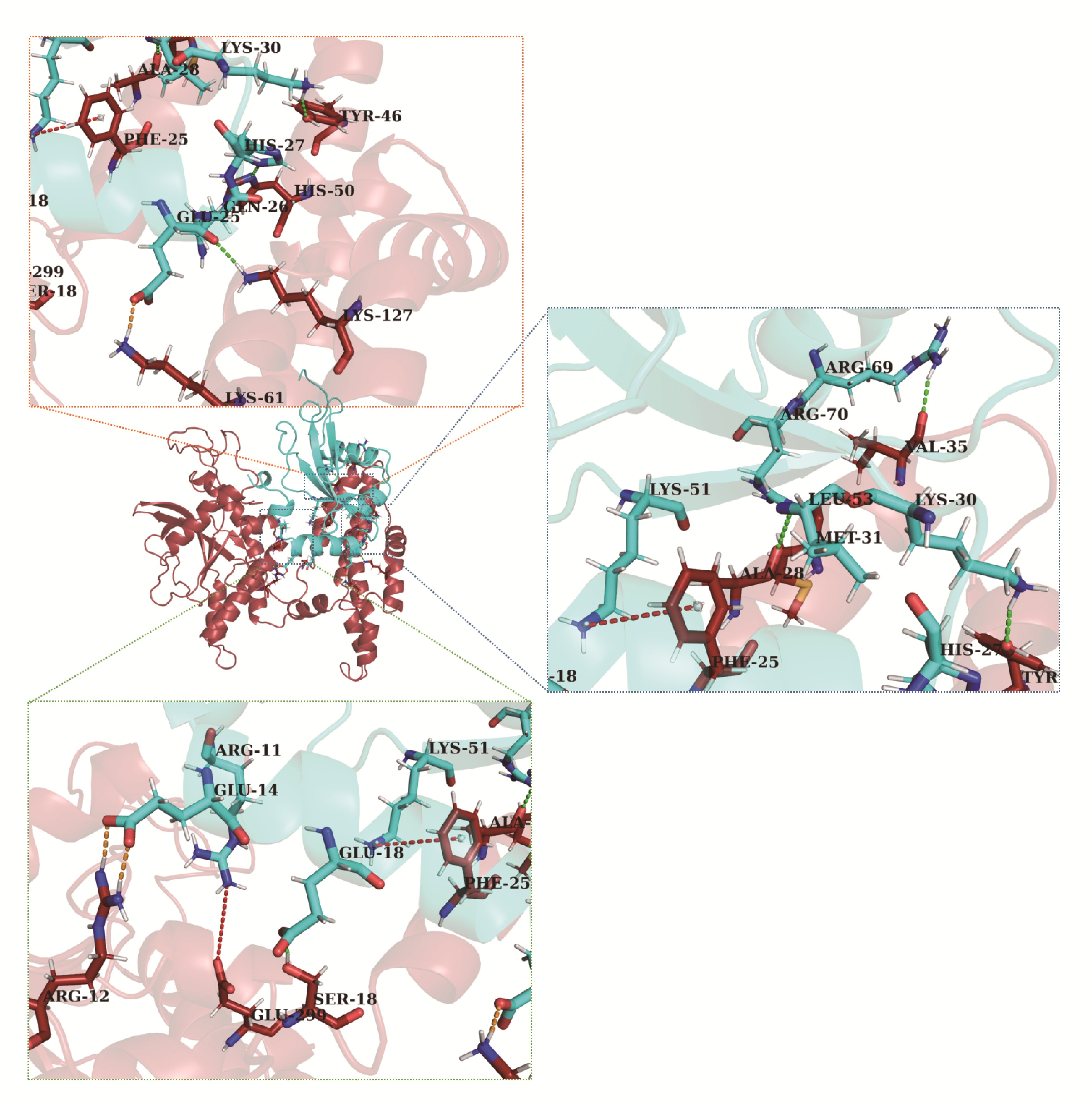

Supplement: Supplementary file 7 — Supplementary Figure 6 [file 41419_2024_7208_MOESM7_ESM.tif]
