# Supplementary material for: STING orchestrates microglia polarization via interaction with LC3 in autophagy after ischemia
Source: Cell Death Dis. 2024 Nov 13;15(11):824. doi: 10.1038/s41419-024-07208-1 (PMC11560960; doi:10.1038/s41419-024-07208-1)
Supplement: Supplementary file 8 — Supplementary table [file 41419_2024_7208_MOESM8_ESM.pdf]

Supplementary Table1 Sequences of primers used in this study.

Gene	Primer sequence
iNOS	FP: AATGCCCGTACCAGGCCCAAT;<br>RP: TAGAGCCCACGCCATCCACTGG
IL-1β	FP: TTGTTCATCTCGGAGCCTGTA;<br>RP: CTACTTCCTTTTCTTCCACGA
TNFα	FP: ATCCGCGACGTGGAACTAG;<br>RP: AAGGTCTTGAGGTCCGCCA
IL-6	FP: GCACTAGGTTTGCCGAGTAGA;<br>RP: GAGGAAGACACTGAGGTCGAA
CD16/32	FP: TTTGGACACCCAGATGTTTCAG;<br>RP: GTCTTCCTTGAGCACCTGGATC
Arg-1	FP: TCTTTGGCAGATATGCAGGGA;<br>RP: CGAAGGTTGACGGTCTGACAC
IL-10	FP: GCCTGGGGCATCACTTCTACC;<br>RP: CAGCCAATCGTCATACAACAGGTC
CD206	FP: AAGGAAGGTTGGCATTTGT;<br>RP: CTTTCAGTCCTTTGCAAGC
GAPDH	FP: AAGAAGGTGGTGAAGCAGG;<br>RP: TGAGGGTGAGAAGGTGGAAG
