# Supplementary material for: STING orchestrates microglia polarization via interaction with LC3 in autophagy after ischemia
Source: Cell Death Dis. 2024 Nov 13;15(11):824. doi: 10.1038/s41419-024-07208-1 (PMC11560960; doi:10.1038/s41419-024-07208-1)

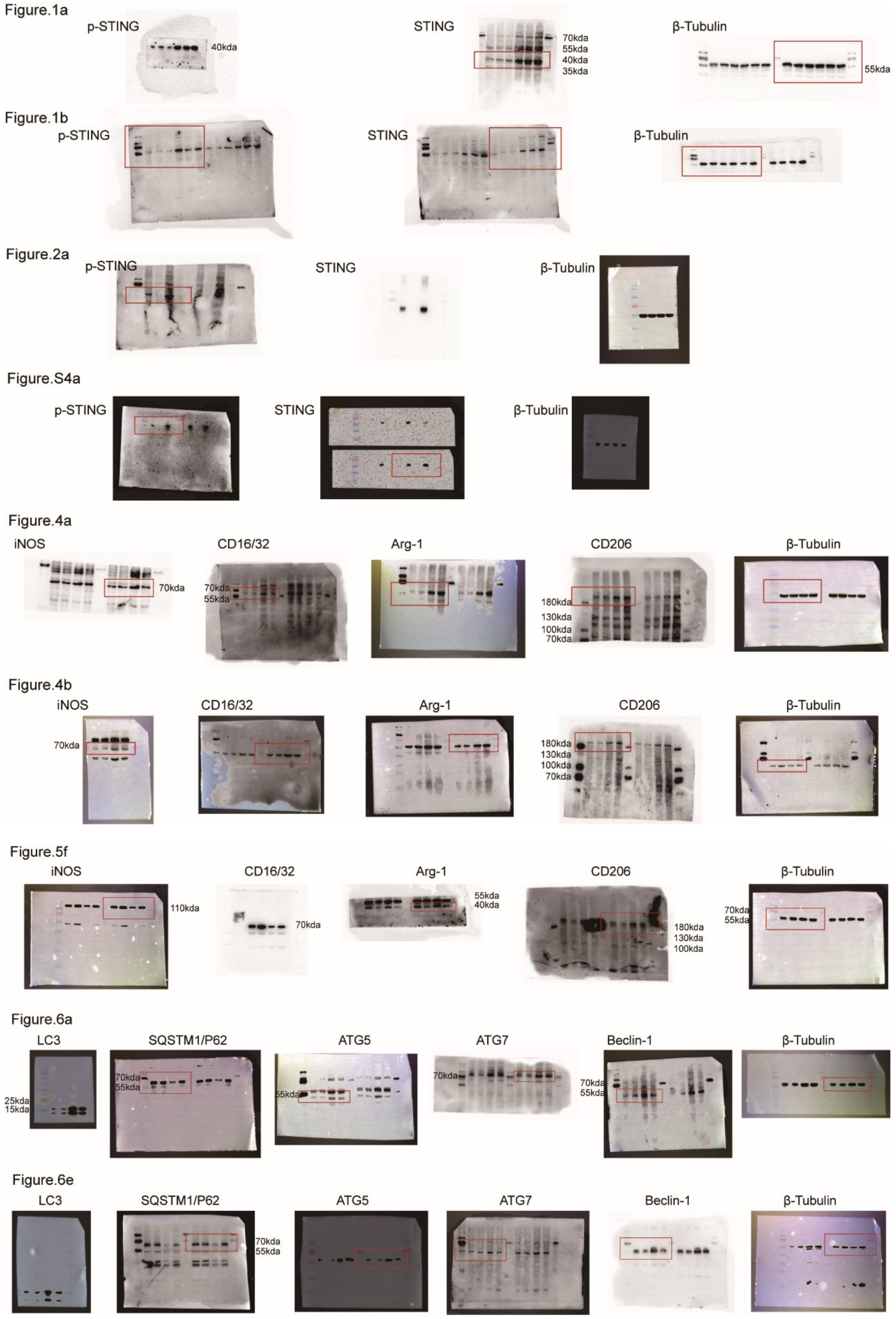

Figure.1a
p-STING
40kda
STING
70kda
55kda
40kda
35kda
β-Tubulin
55kda
Figure.1b
p-STING
STING
β-Tubulin
Figure.2a
p-STING
STING
β-Tubulin
Figure.S4a
p-STING
STING
β-Tubulin
Figure.4a
iNOS
70kda
CD16/32
70kda
55kda
Arg-1
CD206
180kda
130kda
100kda
70kda
β-Tubulin
Figure.4b
iNOS
70kda
CD16/32
Arg-1
CD206
180kda
130kda
100kda
70kda
β-Tubulin
Figure.5f
iNOS
110kda
CD16/32
70kda
Arg-1
55kda
40kda
CD206
180kda
130kda
100kda
β-Tubulin
70kda
55kda
Figure.6a
LC3
25kda
15kda
SQSTM1/P62
70kda
55kda
ATG5
55kda
ATG7
70kda
Beclin-1
70kda
55kda
β-Tubulin
Figure.6e
LC3
SQSTM1/P62
70kda
55kda
ATG5
ATG7
Beclin-1
β-Tubulin

Figure.7c

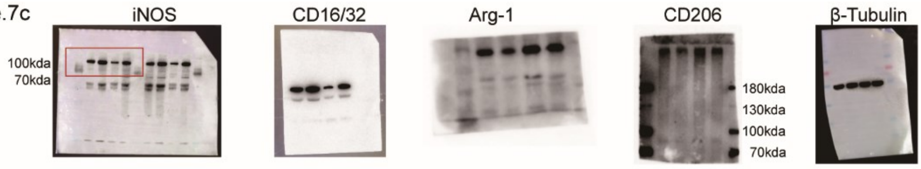

Figure.7d

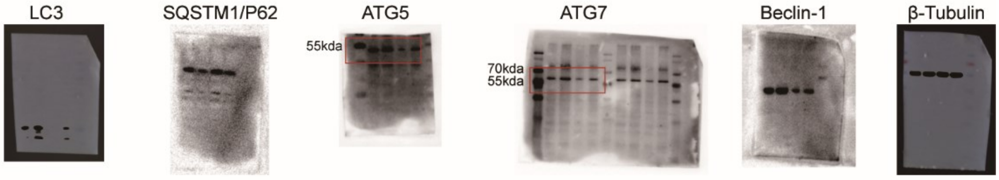

Figure.7h

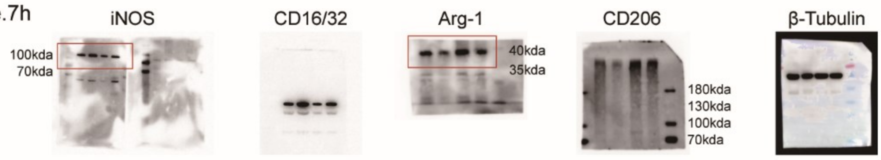

Figure.7i

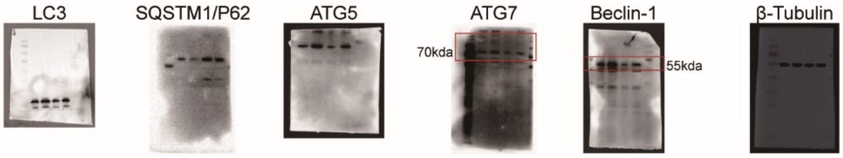

Figure.8a

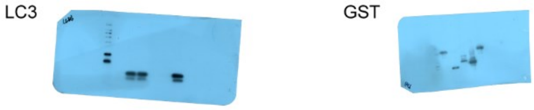

Figure.8b

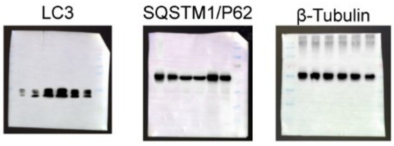

Figure.8c

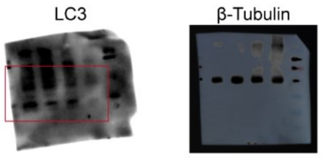

Figure.8e

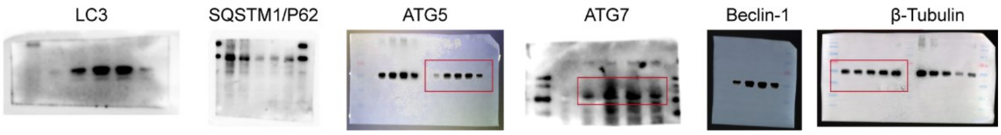

Supplement: Supplementary file 9 — Supplementary materials-original bolts [file 41419_2024_7208_MOESM9_ESM.pdf]
